# Influence of humidity on the initial emittable concentration of formaldehyde and hexaldehyde in building materials: experimental observation and correlation

**DOI:** 10.1038/srep23388

**Published:** 2016-03-30

**Authors:** Shaodan Huang, Jianyin Xiong, Chaorui Cai, Wei Xu, Yinping Zhang

**Affiliations:** 1 Department of Building Science, Tsinghua University, Beijing 100084, China; 2 Beijing Key Lab of Indoor Air Quality Evaluation and Control, Beijing 100084, China; 3 School of Mechanical Engineering, Beijing Institute of Technology, Beijing 100081, China; 4 China Academy of Building Research, Beijing 100013, China

## Abstract

Humidity is one of the main environmental factors affecting the emission rate and key parameters of formaldehyde and volatile organic compounds (VOCs) from building materials. Meanwhile, the initial emittable concentration (*C*_m,0_) is proved to be the most sensitive key parameter to the emission behaviours. However, there is no report on the relationship between humidity and *C*_m,0_. In this paper, *C*_m,0_ of formaldehyde and hexaldehyde from a type of medium density fiberboard in absolute humidity (AH) range of 4.6–19.6 g/m^3^ at 25 °C were tested by virtue of a C-history method. Experimental results indicate that *C*_m,0_ is dramatically dependent on AH, increased by 10 and 2 times for formaldehyde and hexaldehyde when AH rising from 4.6 g/m^3^ to 19.6 g/m^3^. A linear relationship between the logarithm of *C*_m,0_ and AH is obtained based on the measured results. In addition, a correlation characterizing the association of emission rate and AH is derived. The effectiveness of the correlation is verified with our experimental results as well as data from literature. With the correlations, the *C*_m,0_ or emission rate different from the test AH conditions can be conveniently obtained. This study should be useful for predicting the emission characteristics of humidity changing scenarios and for source control.

Poor indoor air quality caused by formaldehyde and volatile organic compounds (VOCs) from building materials may give rise to decreased work efficiency and health-related problems^1^,^2^,^3^,^4^,^5^,^6^,^7^. Short-term exposure to formaldehyde and VOCs results in acute diseases, such as irritating responses of eyes, respiratory symptoms, headache, tiredness and asthma symptoms^1^,^2^,^3^. Study also revealed the adverse effects of formaldehyde exposure on semen quality^4^. Long-term exposure to these pollutants will even cause cancer^5^. The annual output of artificial board in China reached up to about 256 million cubic meters in 2013 and is expected to rising gradually^8^. Therefore, great concern has been posed to the indoor air pollution released from building materials. In order to realize effective control on the indoor formaldehyde and VOCs pollution level, it is urgently needed to understand and predict the source emission behaviours firstly.

The emission behaviours of formaldehyde and VOCs from building materials can be characterized by the emission rate or three key parameters, i.e., the initial emittable concentration (*C*_m,0_), the diffusion coefficient (*D*_m_), and the partition coefficient (*K*). The emission rate or key parameters are not only dependent on the physical properties of the material-pollutant combinations but also affected by the environmental conditions, such as temperature and humidity. The influence of temperature on emission behaviours has been studied by many researchers. For the emission rate, experimental and theoretical studies generally showed an increase of emission rate with temperature^9^,^10^,^11^,^12^,^13^,^14^. While for the key parameters, Zhang *et al.*^15^ established a theoretical correlation between *K* and temperature, Deng *et al.*^16^ derived a correlation between *D*_m_ and temperature, and Huang^17^ proposed a correlation between *C*_m,0_ and temperature for formaldehyde emissions.

The impact of humidity on the emission rate and key parameters is another hot topic besides temperature effect. Former studies mostly focused on the impact of relative humidity (RH) on emission behaviours. Many experimental studies indicated that the emission rate and chamber concentration increased with increasing RH. Andersen *et al.*^9^ observed that the emission rate of formaldehyde from a kind of particleboard was doubled when RH increased from 30 to 70%. Lin *et al.*^11^ reported that when RH increased from 50 to 80%, the emission rate and chamber concentration of toluene, n-butyl acetate, ethylbenzene, and m,p-xylene increased 3.5–5.4, 1.1–1.4, 1.8–3.8, and 1.5–3.5 times, respectively. However, some other studies found that the impact of RH on the emission rate was not always positively correlated. For some scenarios it became ignorable or even negatively correlated for the tested material-pollutant combinations^18^,^19^, and the reason for this phenomenon was unclear. As far as the key parameters are concerned, they are all targeted at the impact of RH on *D*_m_ and *K*. The negligible effect of RH on *D*_m_ is gained by many studies, but things become a little complicated for the impact RH on *K*. Farajollah *et al.*^20^ found that the effect of RH (in the range of 0–40%) on *D*_m_ of tested VOCs could be ignored. Deng^21^ observed in the experiment that the impact of RH on *D*_m_ and K for 6 kinds of VOCs in the tested materials in the RH range of 25–80% was not remarkable. Huang *et al.*^22^ pointed out that RH had no significant impact on *K* for the tested VOCs except for methanol. Xu and Zhang’s^23^ experimental results revealed that: *D*_m_ was insignificant with RH for the tested material-pollutant combination; *K* of formaldehyde increased with the increase of RH in the range of 50–80%, but there was no obvious difference in *K* in the RH range of 25–50%.

There are still some deficiencies in the existing researches. Firstly, available studies are all focused on experimental tests at different relative humidity (RH). The reason for the dependence of emission behaviours on humidity is that the moisture content of the building material changes with humidity. However, the factor directly related with the moisture content is the absolute humidity (AH) rather than the relative humidity in the indoor environment. For pollutant emission tests under constant temperature, the AH can be regarded as equivalent to RH, that is to say, either AH or RH can be used during the test. Nevertheless, if the temperature varies during the emission tests, AH is demonstrated to be a more appropriate parameter according to the field test results^24^. Secondly, there is no report about the influence of humidity on *C*_m,0_, which is proved to be the most sensitive key parameter to the emission behaviours compared with *D*_m_ and *K*^25^,^26^. In addition, there is no correlation between the emission rate and humidity from theoretical studies, which prevents determining the emission rate at humidity different from the test conditions.

Considering that, the main objectives of this paper are to: (1) study the impact of absolute humidity (AH) on *C*_m,0_ for formaldehyde and hexaldehyde emissions from building materials in the AH range of 4.6–19.6 g/m^3^; (2) derive a correlation between the emission rate and AH by virtue of theoretical approach.

## Methods

### Measurement method of the key parameters

For the present study, a rapid and accurate method, the ventilated chamber C-history method^27^, is applied to determine the three key emission parameters (*C*_m,0_, *D*_m_, *K*). The principle of this method is briefly introduced here. The ventilated chamber C-history method comprises two physical processes. The first process is emission under airtight condition. The tested building material is placed in an airtight chamber (air exchange rate, *N* ≈ 0) until the emission process approaches equilibrium. Subsequently, clean air is introduced into the chamber, and this process is the taken as the second process, or, emission under ventilated condition (*N* > 0). Supplementary Fig. S1 (in the Supplementary Information) shows the concentration change tendency of formaldehyde and VOCs in the chamber during these two emission processes.

For the first emission process, according to mass conservation of formaldehyde and VOCs in the gas phase and material phase, the equilibrium chamber concentration can be expressed as:


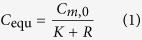


where, *R* is defined as the ratio of chamber volume (*V*_c_) to material volume (*V*_m_).

For the second emission process, by analyzing the mass transfer process in detail and introducing some assumptions, the following equations can be derived^27^:


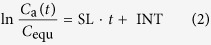



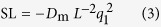



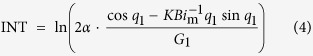


where, *t* is the emission time solely under ventilated condition, s; *C*_a_ is the hourly formaldehyde or VOC concentrations under ventilated condition, μg/m^3^; 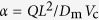; *L* is the half thickness of the material (double surface emission), m; *Q* is the ventilation rate, m^3^/s; *q*_1_ is the first positive root of *q*_*n*_, 
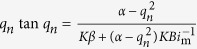

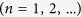
; *G*_1_ is the first term of *G*_*n*_, 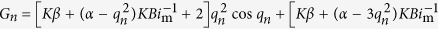

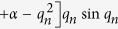
; *Bi*_m_ is the Biot number for mass transfer (=*h*_m_*L*/*D*_m_); *h*_m_ is the convective mass transfer coefficient, m/s, which can be calculated by virtue of empirical correlations^28^.

Once the experimental data of equilibrium concentration (*C*_equ_) under airtight condition and a group of hourly concentrations (*C*_a_) under ventilated condition are measured, and then substituted into equation (2), slope (SL) and intercept (INT) of equation (2) can be determined from linear curve fitting. The two parameters, *D*_m_ and *K*, which are functions of SL and INT, can be conveniently obtained by solving the two equations. Then, by combining the determined *K* with equation (1), *C*_m,0_ can be calculated. For the ventilated chamber C-history method, although the three key parameters are obtained simultaneously, the solutions are unique from mathematics because the principle of this method is to solve three equations containing the three parameters.

### Experiment

**Figure 1:**
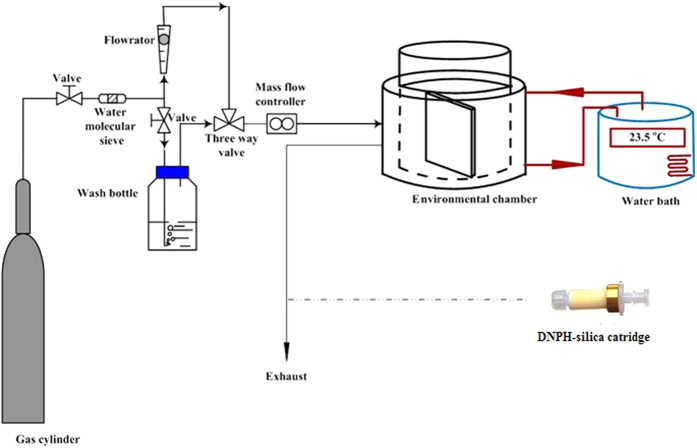
Schematic of the experimental system.

The experimental system for determining the key emission parameters under different AHs is schematically shown in Fig. 1. It is comprised of a 30 L stainless steel chamber, a temperature control system, a humidity control system and a sampling system. A fan is fixed in the top of the chamber to accelerate the mixing of pollutants in the air phase. In addition, a stainless steel shelf is placed inside the chamber to fix the tested material. The temperature control system includes a water bath, an annular cavity formed by the inside and outside walls of the chamber. Absolute humidity is controlled by a triple valve with three entrances to clean air, water vapor and the chamber air. The sampling system consists of a sampling pump linked with a DNPH (dinitrophenylhydrazone)-silica cartridge for aldehydes analysis.

A kind of medium density fiberboard (MDF) with thickness of 3 mm was chosen for test, which is widely used for decoration in China. Moreover, it is very rapid for this kind of MDF (thin) to reach steady state under both airtight and ventilated conditions, so as to shorten the test time. The MDF purchased from the market was cut into small pieces, with length and width of 10.0 cm × 10.0 cm. The edge of the MDF was wrapped with foil, thus just the front and back of the board were exposed to air during the test.

Two kinds of aldehydes, formaldehyde and hexaldehyde, were chosen as the target pollutants. Formaldehyde was selected because it is classified as a human carcinogen and the main pollutant emitted from wood-based boards. The selection of hexaldehyde was due to that it is a typical aldehyde causing health hazard indoors. Moreover, these two aldehydes were detectable and showed relatively high concentrations during the whole test, but the concentrations of other aldehydes were very low and thus were not appropriate for analysis with the ventilated chamber C-history method due to the sparse data and low measurement accuracy. At the beginning, the chamber maintains airtight until the material emission process reaches equilibrium. Subsequently, clean air is introduced into the chamber. For the airtight scenario, the equilibrium time for some common material-pollutant combinations is no more than 24 h. For the sake of safety, the MDF is pretreated under airtight condition for 36 h. Once equilibrium is arrived, the equilibrium gas phase concentration is sampled for one time, and then clean air with flow rate of 5.0 L/min is supplied into the chamber. For the ventilated condition tests, chamber air is sampled for several times at regular intervals, with sampling rate of 0.2 L/min and sampling time of 5 min. The sampled air is then pulled through a DNPH-silica cartridge. Duplicate samplings are taken at one time in order to reduce measurement error. All the sampled cartridges are kept in a cold refrigerator until analyzed by HPLC (Agilent 1260), which is a widely used measurement technique for carbonyl compounds, i.e., formaldehyde, other aldehydes and ketones. When analyzing, the cartridge was eluted with about 20 ml of acetonitrile for 15 min in an ultrasonic cleaner and then shocked in vortex mixer for 30 s. After that it was passed through a needle type filter. 20 μl of the solution obtained after extraction were injected by an automatic sampler into a reversed phase column C_18_. The operating condition of HPLC is as follows: chromatographic column: Welch Ultimate XB-C18; mobile phase: acetonitrile-water (60/40); velocity of flow: 1.5 ml/min; detection wavelength: 360 nm; column temperature: 30 °C; sample size: 20 μl. The total test time under the ventilated condition is generally no more than 12 h.

In order to investigate the relationship between *C*_m,0_ and AH, the experiments for determining key parameters are carried out under 5 different AHs, i.e., 4.6 ± 0.5 g/m^3^, 9.2 ± 0.5 g/m^3^, 12.7 ± 0.5 g/m^3^, 15.0 ± 0.5 g/m^3^ and 19.6 ± 0.5 g/m^3^. The air temperature in the chamber is controlled at 25.0 ± 0.5 °C. The geometrical dimensions of the tested building material and experimental conditions are listed in Table 1.

In the previous section, we pointed out that the emission characteristics of pollutants from building materials is dependent on the moisture content of the materials, which is related with AH only when the water vapor reaches equilibrium at a certain temperature. Generally, the building material may adsorb/desorb water vapor from/to the air phase of the chamber when it is put into the chamber, which means that the moisture content of the building material is not stable during the initial emission stage. Since the emission behaviors varies with the change of moisture content, it is meaningful to measure the key emission parameters only when the water vapor reaches equilibrium (moisture content doesn’t change). We analyze the time to reach equilibrium for two kinds of chamber studies: one is the widely used ventilated chamber experiment, and the other is the experimental procedure used in this study (firstly airtight then ventilated). With the *K* of water vapor measured (2894) and *D*_m_ taken from reference^29^, the change of AH in the traditional ventilated chamber test is simulated, and the results are shown in Fig. S2. This figure indicates that the time to reach water vapor equilibrium is quite long (about 40 h), meaning that the traditional chamber method will cause measurement error if the samplings were taken in 40 h. We also simulate the change of AH in the chamber during the airtight procedure of the ventilated C-history method. Figure S3 shows the results with the equilibrium AH value of 12.7 g/m^3^. This figure reveals that it just takes about 0.2 h for the AH to reach a stable level (adsorb water vapor), implying that the moisture content in the building material reaches equilibrium after 0.2 h, which is much shorter than the whole airtight procedure (36 h). After 36 h, it is the ventilated procedure. The AH of air introduced into the chamber is controlled to approximate the equilibrium value in the airtight procedure. For the ventilated chamber C-history method, we only need to take samplings at the end of the airtight procedure and at regular intervals of the ventilated procedure. According to the above analysis, the moisture content of building material is stable when we take samplings. Another experiment indicates that the moisture content in the building material is in a good linear relationship with AH when the water vapor reaches equilibrium for the present method, as shown in Fig. S4.

In order to ensure the accuracy and precision of the experiment results, we also did recovery rate experiments with a standard formaldehyde reference^30^. The experimental results of recovery rate in a ventilated chamber is shown in Table S1. The average recovery rate and the relative standard deviation (RSD) are 85.6% and 3.3% (n = 6) respectively, demonstrating good accuracy of the experiment^31^.

## Results and Discussion

### Determination of the key parameters at different AHs

**Figure 2:**
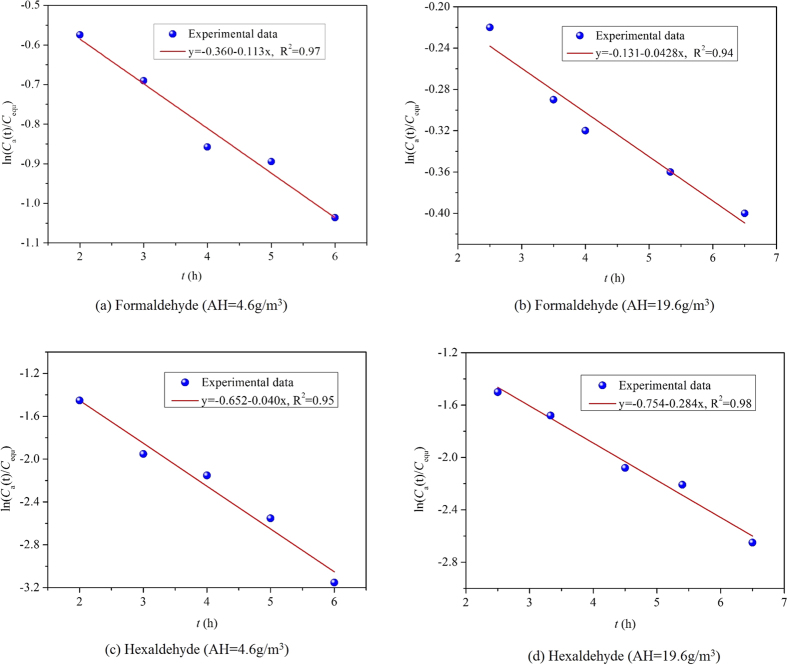
Linear relationship between ln(*C*_a_/*C*_equ_) and time at AH of 4.6 g/m^3^ and 19.6 g/m^3^ by fitting the experimental data.

Duplicate samplings are performed at each time, and the average value of the two samplings is taken as the chamber concentration (*C*_a_). By applying equation (2), the linear curve fitting results of the experimental data at two AHs, 4.6 g/m^3^ and 20.0 g/m^3^, are shown in Fig. 2. The results at other AHs are included in Supplementary Fig. S5. According to Fig. 2 and Fig. S5, all the squares of the correlation coefficients (R^2^) of regressions are over 0.90, indicating good measurement accuracy^32^.

**Figure 3:**
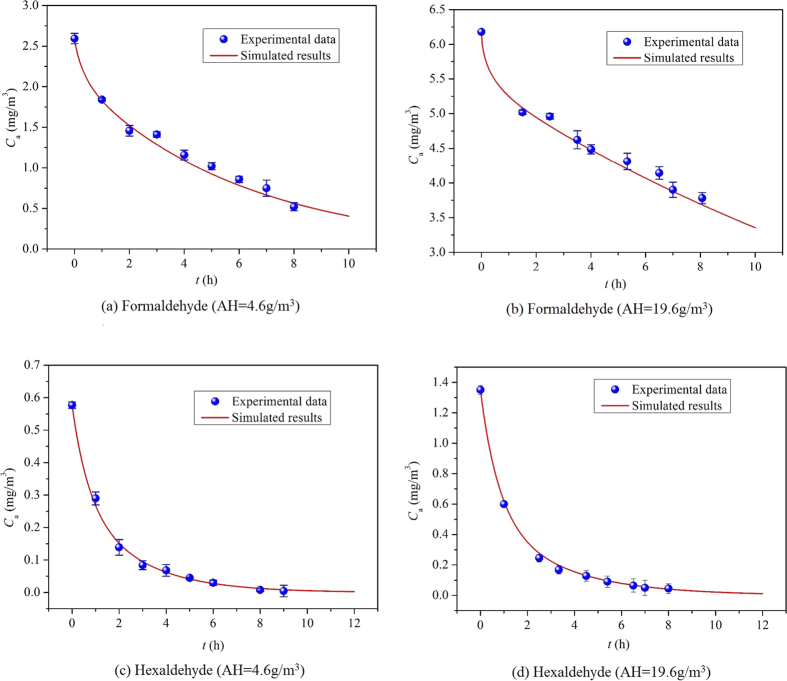
Comparison of chamber formaldehyde concentration between the simulated results and experimental data at AH of 4.6 g/m^3^ and 19.6 g/m^3^.

Based on the SL and INT of the regression line, the three key emission parameters (*C*_m,0_, *D*_m_, *K*) of formaldehyde and hexaldehyde at the different AHs can be determined by virtue of the ventilated chamber C-history method. Table 2 summarizes the measured key parameters of formaldehyde and hexaldehyde and the corresponding R^2^ at 5 different AHs. The determined parameters should be validated to prove its reliability. As a preliminary validation, we calculate the chamber formaldehyde concentration under the ventilated condition using an analytical model^33^ based on the determined parameters given in Table 2, and then compare it with the experimental data. Figure 3 shows the comparison between the simulated results and experimental data at AH of 4.6 g/m^3^ and 19.6 g/m^3^, while Supplementary Fig. S6 shows the comparison at other AHs. Good agreements between the simulations and experiments in these two figures demonstrates the effectiveness of the measured parameters.

Figure 3 and Fig. S6 further indicate that the decay of hexaldehyde concentration is much faster than that of formaldehyde at the same AH. According to the results from sensitive analysis^27^, *K* significantly affects the chamber pollutant concentration at the initial period. As shown in Table 2, at the same AH, *K* of formaldehyde is about 10 times of that of hexaldehyde, which leads to the more rapid decay of hexaldehyde concentration in Fig. 3 and Fig. S6.

### Impact of AH on *C*_m,0_

According to the determined *C*_m,0_ listed in Table 2, with the increase of AH, *C*_m,0_ of both formaldehyde and hexaldehyde increases greatly. For formaldehyde, *C*_m,0_ at AH of 4.6 g/m^3^ is 2.07 × 10^6^ μg/m^3^; when AH increases to 19.6 g/m^3^, *C*_m,0_ becomes 2.39 × 10^7^ μg/m^3^, which is about 11 times of that at 4.6 g/m^3^. Similarly, *C*_m,0_ of hexaldehyde at AH of 19.6 g/m^3^ is about 3 times of that at 4.6 g/m^3^. It is evident that the impact of AH on *C*_m,0_ of formaldehyde is much more significant than that of hexaldehyde.

**Figure 4:**
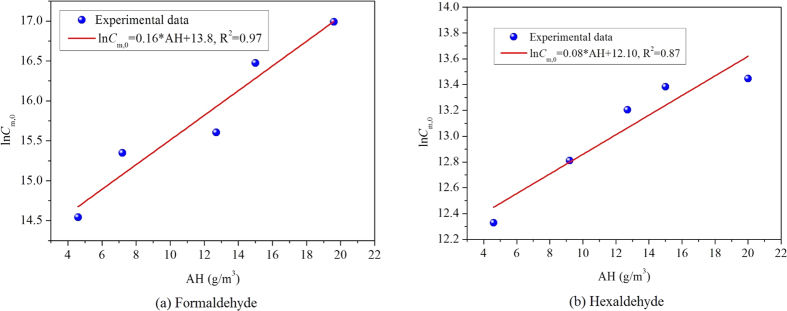
The relationship between logarithm of *C*_m,0_ and AH for the target aldehydes in MDF.

By analyzing *C*_m,0_ at different AHs in Table 2 in detail, we find that the change tendency between *C*_m,0_ and AH is not simply linear. After taking logarithm of *C*_m,0_ at different AHs, the association between ln *C*_m,0_ and AH is depicted in Fig. 4. This figure shows that ln *C*_m,0_ is in a good linear relationship with AH for both formaldehyde and hexaldehyde. By performing linear curve fitting, the R^2^ for formaldehyde and hexaldehyde are 0.97 and 0.87, respectively, which are relatively high thus proving the linear association between logarithm of *C*_m,0_ and AH.

Based on the above analysis, the correlation between *C*_m,0_ and AH for formaldehyde and hexaldehyde in the tested MDF can be expressed as:


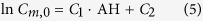


where, *C*_1_, *C*_2_ are constants, which are not dependent on AH but are only related with the physical properties of the material-pollutant combinations. Once two or more sets of experimental data are available to determine the parameters *C*_1_ and *C*_2_ in equation (5), this correlation can then be applied to evaluate *C*_m,0_ at other AHs, which is very useful.

### Impact of AH on *D*_m_

Table 2 indicates that, for the tested AH range, the maximum of *D*_m_ of formaldehyde is 1.49 × 10^−10^ m^2^/s, while the minimum value is 8.57 × 10^−11^ m^2^/s. It means, *D*_m_ of formaldehyde just varies within 43% when AH changes in the range of 4.6 g/m^3^ to 19.6 g/m^3^. Meanwhile, *D*_m_ of hexaldehyde all lies in the magnitude of 10^−10^ m^2^/s during the tested AH range, with relative deviation no more than 49%. Supplementary Fig. S7 shows the change tendency of ln *D*_m_ with AH. A conclusion can be drawn from this figure that *D*_m_ doesn’t change obviously with AH. According to the sensitive analysis in recent study^27^, when *D*_m_ varies in the above fluctuation range, there is no distinctive influence on the emission characteristics. Therefore, the impact of AH on *D*_m_ can be regarded as negligible. This result is similar with the previous studies^20^,^21^,^23^.

### Impact of AH on *K*

The measured parameters in Table 2 imply that *K* of both formaldehyde and hexaldehyde are positively correlated with AH. When AH increases from 4.6 g/m^3^ to 19.6 g/m^3^, *K* increases by 2.3 and 0.7 times for formaldehyde and hexaldehyde, respectively. Obviously, *K* of hexaldehyde changes less than that of formaldehyde, and the reason may be due to that: formaldehyde is water-soluble, which will result in a large *K* when AH increases, while hexaldehyde is insoluble in water. Therefore, the influence degree of AH on *K* is related with the physical properties of the pollutant.

**Figure 5:**
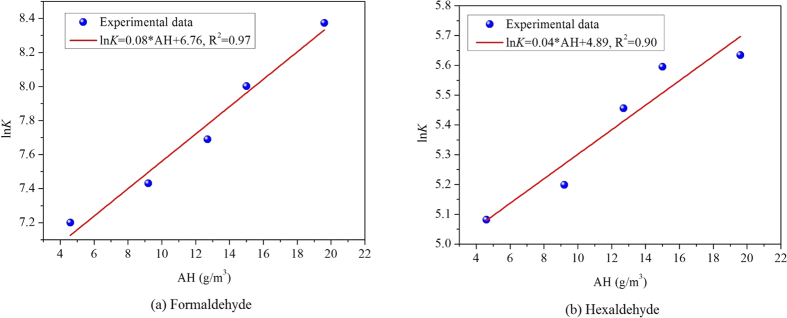
The relationship between logarithm of *K* and AH for the target aldehydes in MDF.

The logarithm of *K* with AH of the two aldehydes is diagrammed in Fig. 5, and a linear association between them is observed. Based on this figure, a correlation between *K* and AH can be obtained:


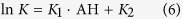


where, *K*_1_, *K*_2_ are constants, which are irrelevant with AH but are only related with the physical properties of the material-pollutant combinations.

### Impact of AH on emission rate

For the impact of AH on the emission rate, traditional studies are all based on experimental investigations. In this section, we devote to derive a theoretical correlation based upon the aforementioned results. When the emissions of formaldehyde and VOCs from building materials reach steady state, i.e., *Fo*_m_ (=*D*_m_*t*/*L*^2^) is no less than 0.2, the emission rate can be expressed as follows by taking dimensionless analysis and simplifications^14^,^34^:


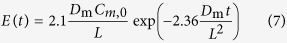


where, *E* is the emission rate factor, μg/(m^2.^h).

According to the results in the present and traditional studies, AH has no obvious impact on *D*_m_ for a certain material-pollutant combination. Therefore, the term 2.1*D*_m_/*δ* can be regarded as a constant irrelevant with AH and equation (7) can be written as:


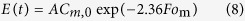


where, *A* = 2.1*D*_m_/*δ*.

Taking logarithm on both sides of equation (8), it yields:


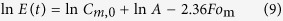


Combining this equation with equation (5), we get:


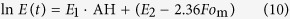


where, *E*_1_ = *C*_1_, *E*_2_ = ln *A* + *C*_2_.

Considering that *Fo*_m_ is approximately 0.2 when the emissions reach steady state and is taken as 2.0 when the emissions are completed^34^,^35^, the term 2.36*Fo*_m_ is relatively small compared with other terms (e.g., *C*_2_, as shown in Fig. 5) in equation (10) and thus can be ignored. The deviation caused by neglecting this term can be reduced by adjusting the parameters *E*_1_ and *E*_2_. Therefore, equation (10) can be further simplified as:


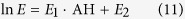


According to the derivation process, the parameters *E*_1_ and *E*_2_ in this equation are independent on AH. Equation (11) indicates that the logarithm of the emission rate is linearly associated with AH. Once the steady state emission rate at two or more different AHs are measured, *E*_1_ and *E*_2_ can be determined by solving equations or linear curve fitting, then the emission rate at a certain AH different from the test conditions can be calculated by virtue of the derived correlation (equation (11)).

**Figure 6:**
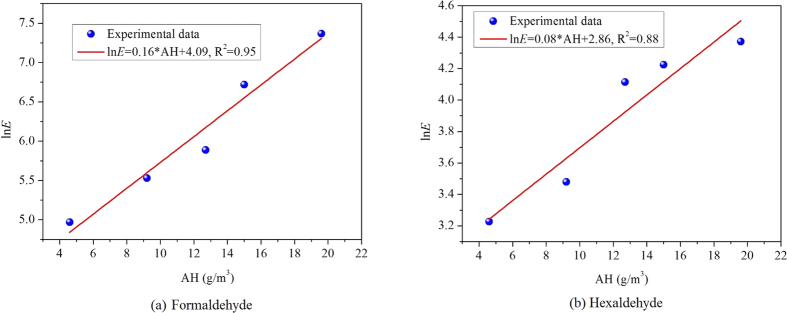
Validation of the derived correlation for emission rate with experimental data of formaldehyde and hexaldehyde from MDF.

The experimental data of formaldehyde and hexaldehyde emissions from MDF is applied to validate the derived correlation. Theoretically, the emission rate is a function of the three key parameters (*C*_m,0_, *D*_m_, *K*). In previous section, the key parameters for formaldehyde and hexaldehyde are determined at different AHs. Therefore, we can calculate the steady state emission rate by applying an analytical model^33^. Figure 6 shows the calculated results (*t* = 40 h) at 5 different AHs (4.6 g/m^3^, 9.2 g/m^3^, 12.7 g/m^3^, 15.0 g/m^3^, and 19.6 g/m^3^). It reveals that ln *E* changes linearly with AH, with R^2^ being 0.97 and 0.87, implying the derived correlation for emission rate is effective and reliable.

The experimental data in literature for other material-pollutant combinations are also taken to further validate the derived correlation. Fang *et al.*^36^ tested the emission rate of several pollutants from different materials at various humidity under different temperatures. Sidheswaran *et al.*^37^ measured the emission rate of formaldehyde from different filters at different humidity under high wind speed and low wind speed. By using the correlation (equation (11)) to treat the experimental data, the results are shown in Supplementary Fig. S8. Supplementary Table S2 gives the detailed regression information. Fig. S8 and Table S2 reveal that, when other environmental conditions are fixed, ln *E* confirms a good linear relationship with AH.

## Conclusions

This paper investigates the impact of absolute humidity on the initial emittable concentration (*C*_m,0_) of formaldehyde and hexaldehyde emissions from one kind of medium density fibreboard. Experimental results indicate that *C*_m,0_ of both aldehydes changes significantly with AH in the range of 4.6–19.6 g/m^3^. A linear association between logarithm of *C*_m,0_ and AH is obtained via regression of the tested results. For the other two key parameters, it is observed that *D*_m_ is irrelevant with AH while *K* is positively related with AH for the two aldehydes in the tested MDF. Furthermore, a novel correlation to describe the relationship between the emission rate and AH is derived theoretically, which is more advantageous than the traditional studies focused on experimental exploration. The accordance between the correlation predictions and experimental data in the present study and literature suggests it will be effective and reliable for evaluating the formaldehyde and VOC emissions from building materials under varied AH conditions. Future study will focus on the comprehensive impact of temperature and AH on the emission characteristics as well as the associated health risks.

## Additional Information

**How to cite this article**: Huang, S. *et al.* Influence of humidity on the initial emittable concentration of formaldehyde and hexaldehyde in building materials: experimental observation and correlation. *Sci. Rep.* **6**, 23388; doi: 10.1038/srep23388 (2016).

## Supplementary Material

Supplementary Information

## Figures and Tables

**Table 1:** Experimental conditions of the tested building material.

AH (g/m^3^)	Temperature (°C)	Dimensions(cm × cm × cm)	Number of pieces
4.6 ± 0.5	25 ± 0.5	10.0 × 10.0 × 0.3	4
9.2 ± 0.5			
12.7 ± 0.5			
15.0 ± 0.5			
19.6 ± 0.5			

**Table 2:** Determined key emission parameters based on ventilated chamber C-history method at 5 different AHs.

AH (g/m^3^)	*C*_m,0_ (μg/m^3^)	*D*_m_ (m^2^/s)	*K*	R^2^
Formaldehyde				
4.6 ± 0.5	2.07 × 10^6^	8.57 × 10^−11^	1.34 × 10^3^	0.90
9.2 ± 0.5	4.63 × 10^6^	1.49 × 10^−10^	2.06 × 10^3^	0.99
12.7 ± 0.5	5.98×10^6^	1.10 × 10^−10^	3.04 × 10^3^	0.98
15.0 ± 0.5	1.43 × 10^7^	1.31 × 10^−10^	3.61 × 10^3^	0.98
20.0 ± 0.5	2.39 × 10^7^	1.14 × 10^−10^	4.33 × 10^3^	0.94
**Hexaldehyde**				
4.6 ± 0.5	2.26 × 10^5^	1.63 × 10^−10^	1.61 × 10^2^	0.93
9.2 ± 0.5	3.66 × 10^5^	2.27 × 10^−10^	1.81 × 10^2^	0.93
12.7 ± 0.5	5.42 × 10^5^	1.17 × 10^−10^	2.34 × 10^2^	0.98
15.0 ± 0.5	6.49 × 10^5^	1.86 × 10^−10^	2.69 × 10^2^	0.93
20.0 ± 0.5	6.91 × 10^5^	1.16 × 10^−10^	2.80 × 10^2^	0.98
