# Supplementary material for: Influence of humidity on the initial emittable concentration of formaldehyde and hexaldehyde in building materials: experimental observation and correlation
Source: Sci Rep. 2016 Mar 30;6:23388. doi: 10.1038/srep23388 (PMC4812241; doi:10.1038/srep23388)
Supplement: Supplementary Information [file srep23388-s1.doc]

**Supplementary Information**

**Manuscript title:**

Influence of humidity on the initial emittable concentration of formaldehyde and hexaldehyde in building materials: experimental observation and correlation

**Author lists:**

Shaodan Huang^1,2^, Jianyin Xiong^3^, Chaorui Cai^1,2^, Wei Xu^4^, Yinping Zhang^1,2,*^

**Fig. S1.** Changes of the chamber formaldehyde and VOC concentrations during the airtight and ventilated processes.

**Fig. S2.** Change of AH in the chamber with the traditional ventilated chamber method with the equilibrium value of 12.7g/m^3^.

**Fig. S3.** Change of AH in the chamber during airtight condition with the equilibrium value of 12.7g/m^3^.

**Fig. S4.** The relationship between the moisture content in the building material and AH.

**Fig. S5.** Linear relationship between ln(*C*_a_/*C*_equ_) and time at AH of 9.2g/m^3^, 12.7g/m^3^ and 15.0g/m^3^ by fitting the experimental data.

**Fig. S6.** Comparison of chamber formaldehyde and hexaldehyde concentration between the simulated results and experimental data at AH of 9.2g/m^3^, 12.7g/m^3^ and 15.0g/m^3^.

**Fig. S7.** The relationship between *D*_m_ and AH for the target aldehydes in MDF.

**Fig. S8.** Validation of the derived correlation for emission rate with experimental data from literature.

Fig. S1.


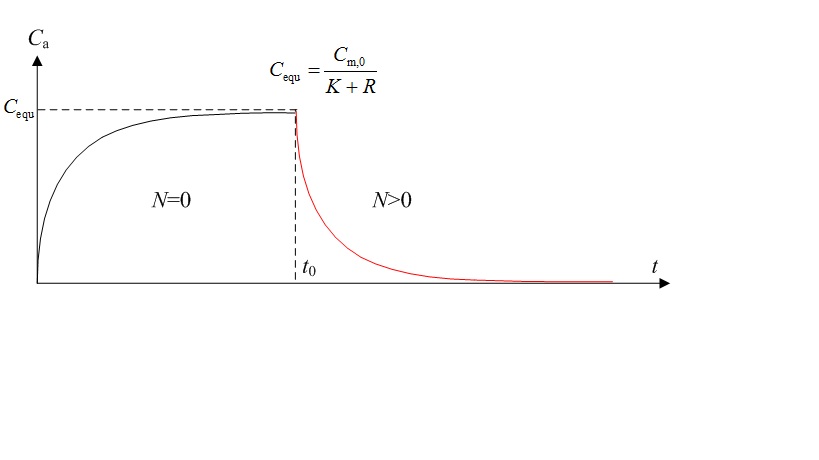


Fig. S2.


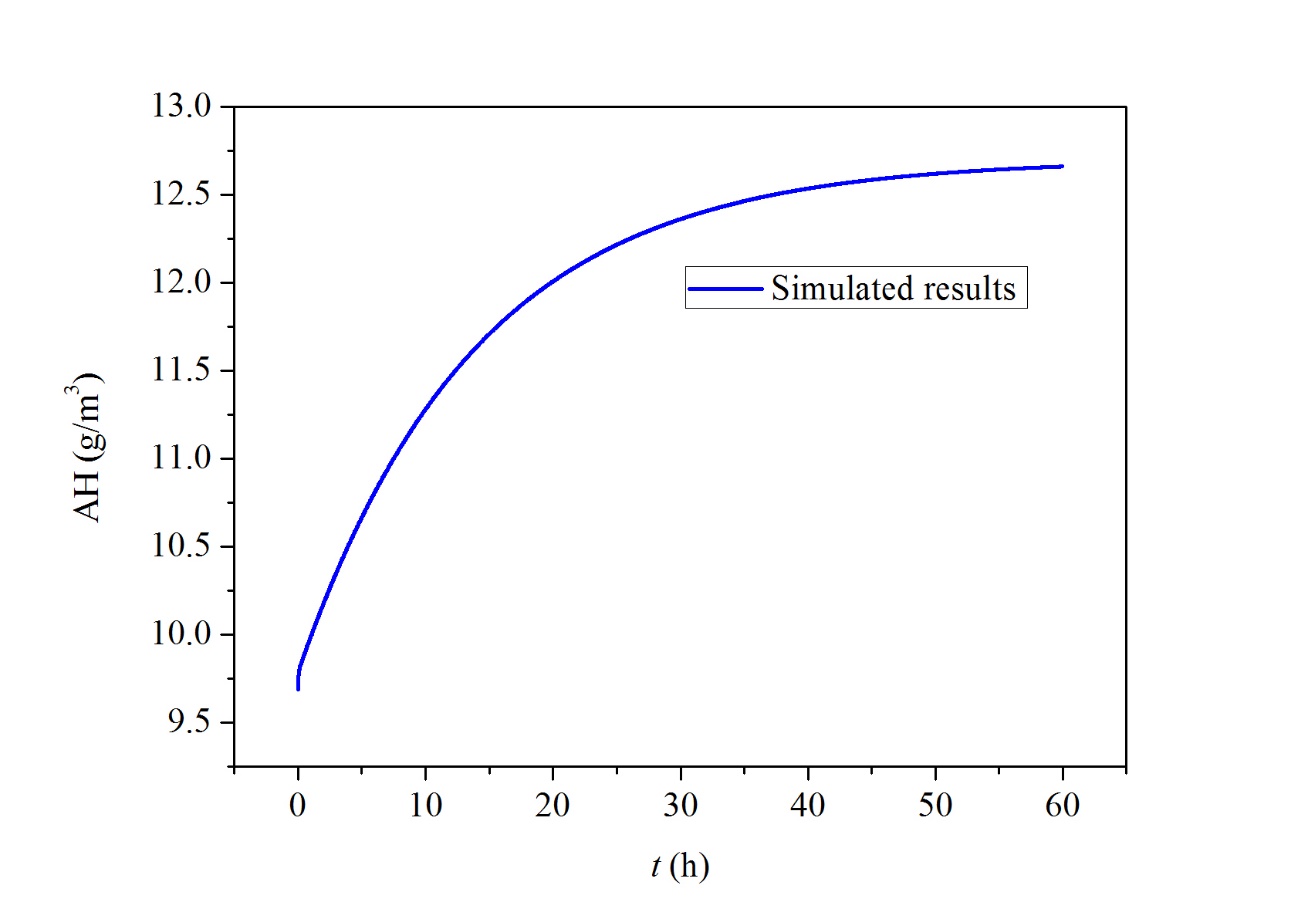


Fig. S3.


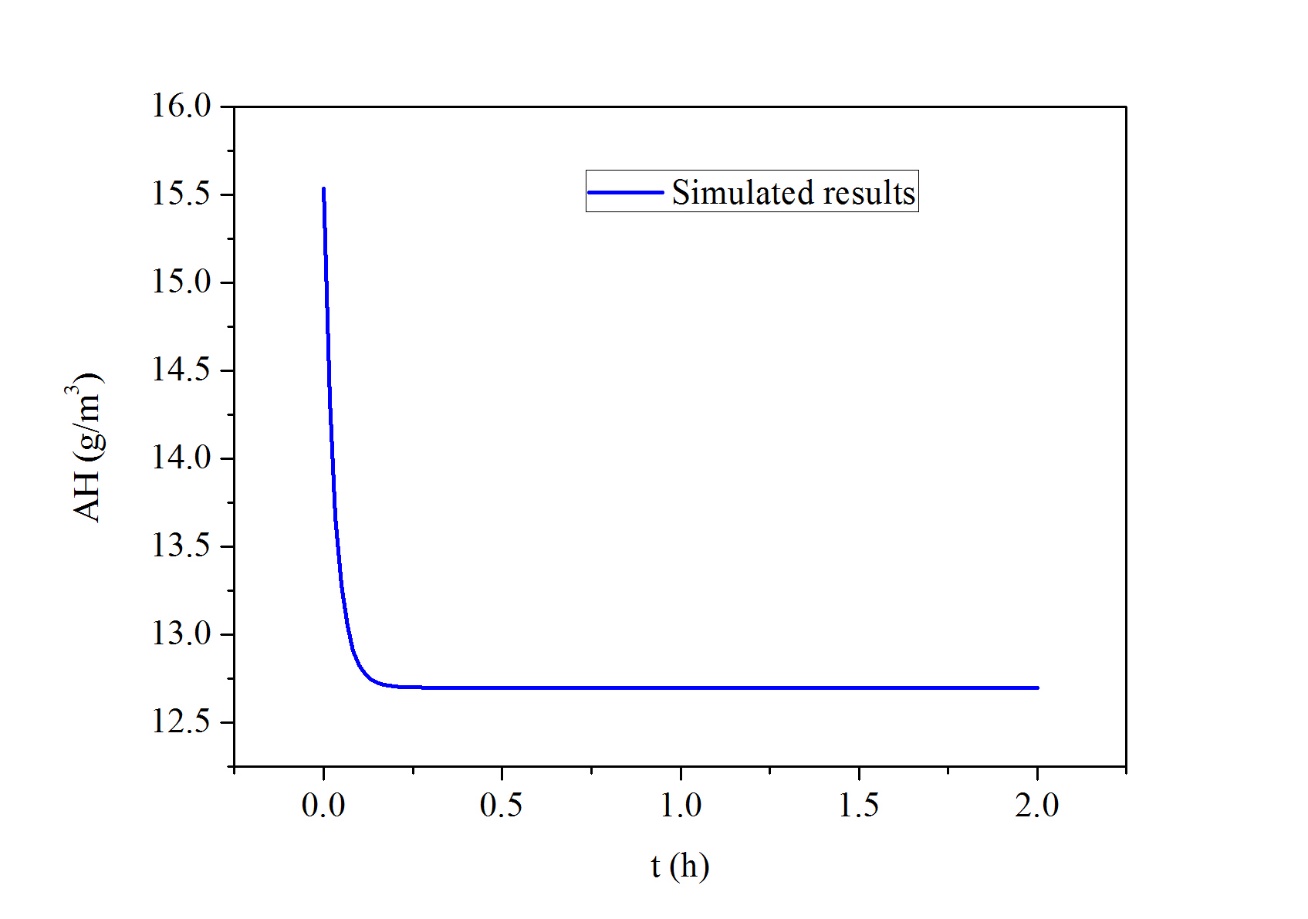


Fig. S4


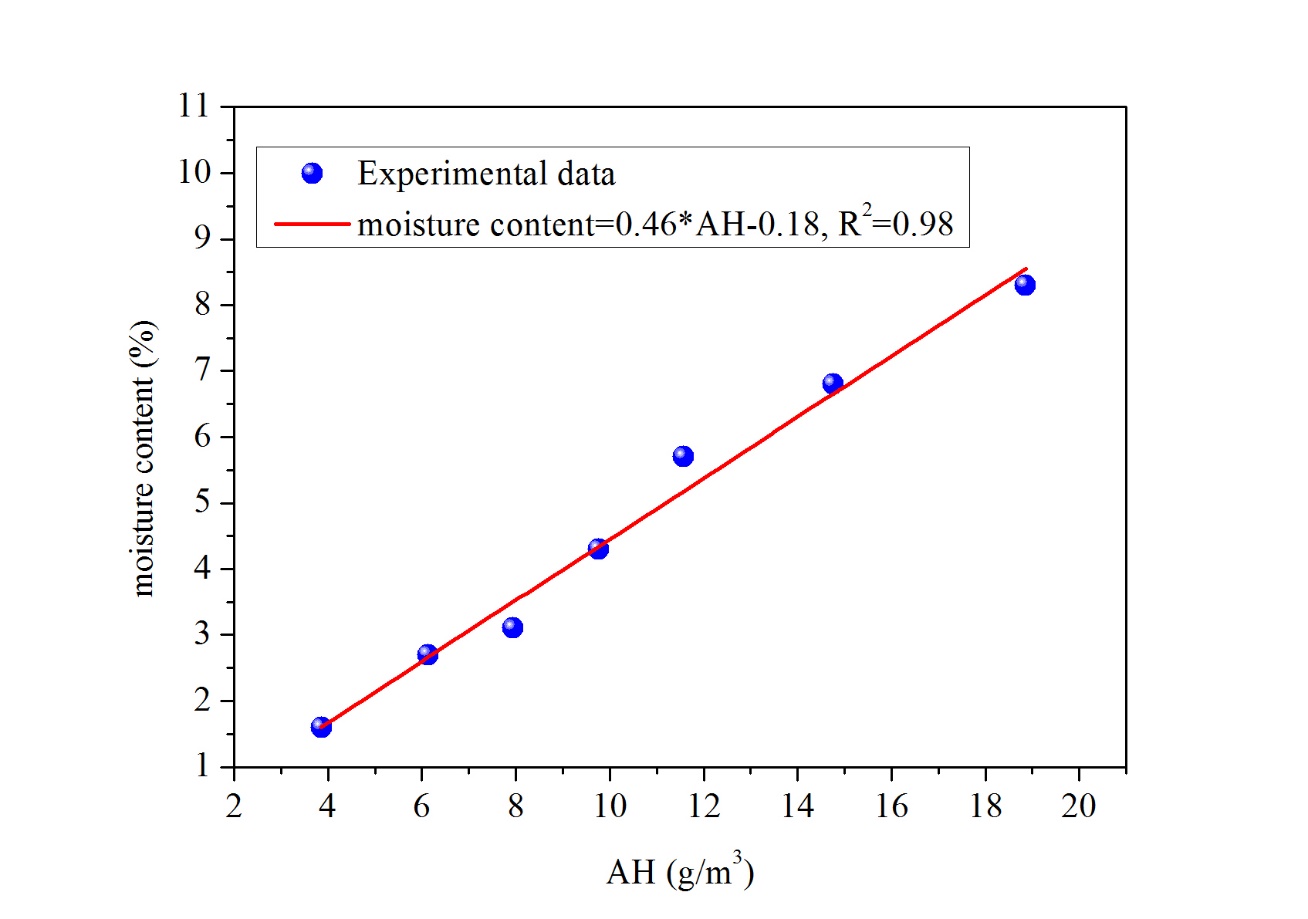


Fig. S5.


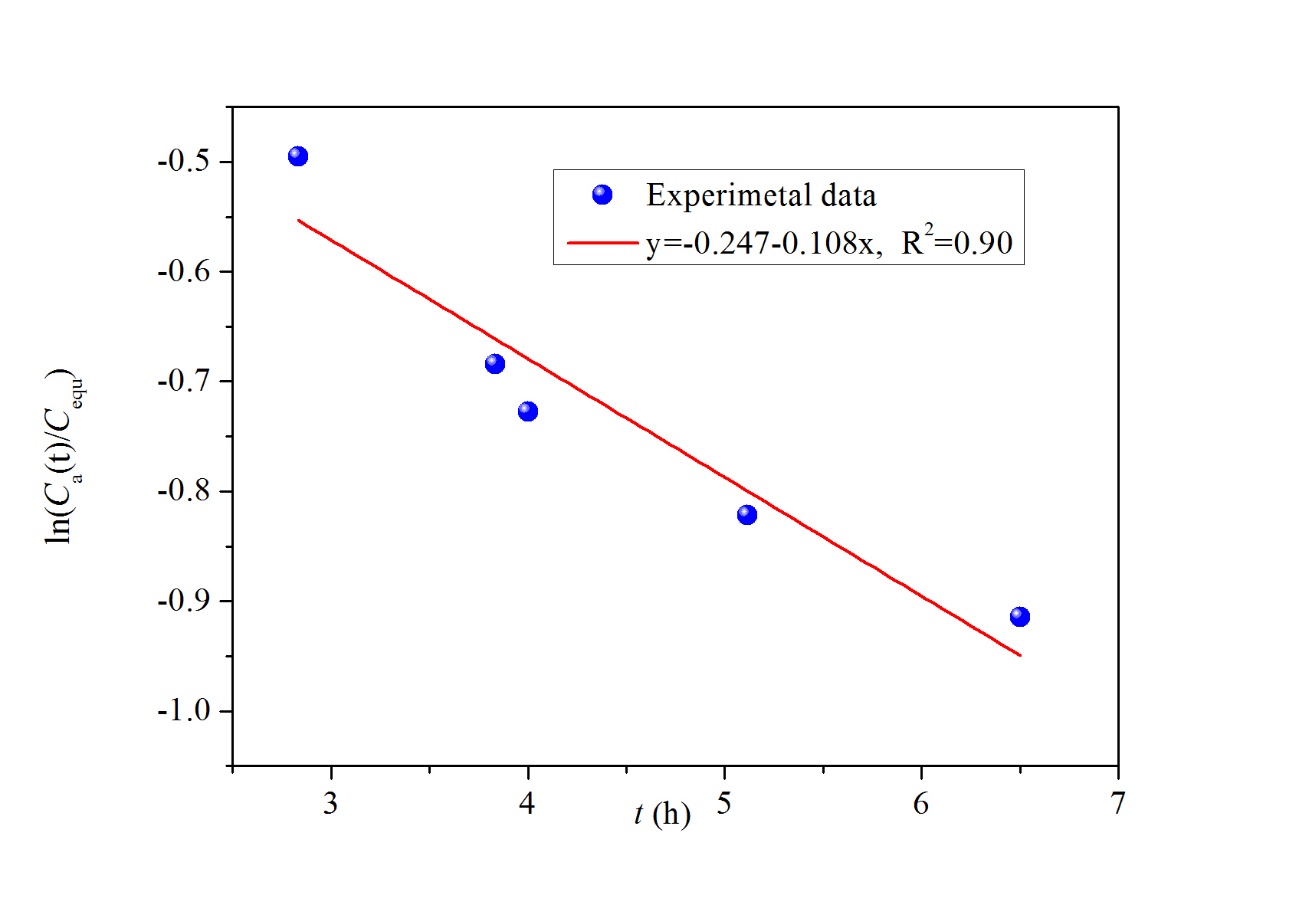


1. Formaldehyde (AH=9.2g/m^3^)


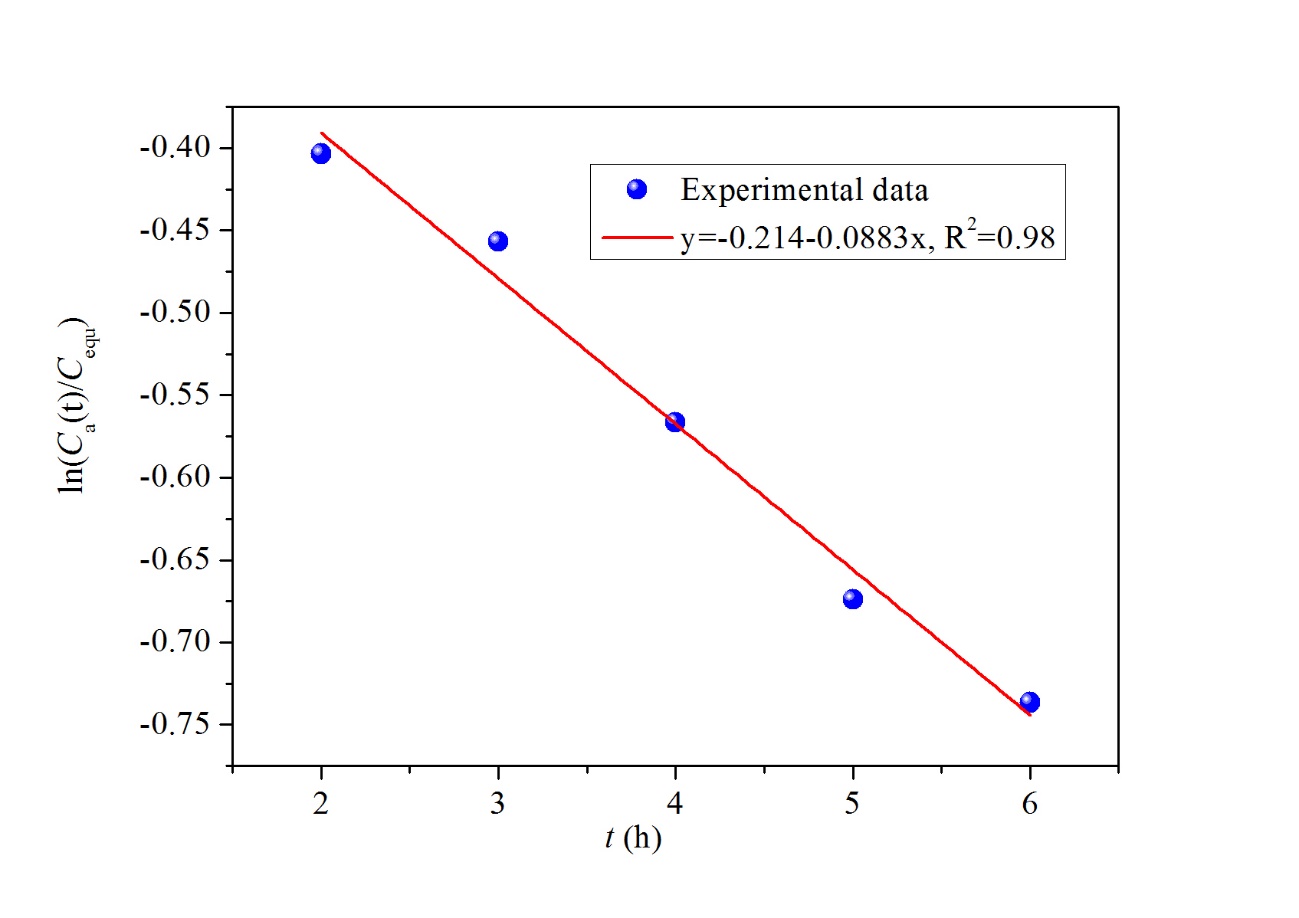


1. Formaldehyde (AH=12.7g/m^3^)


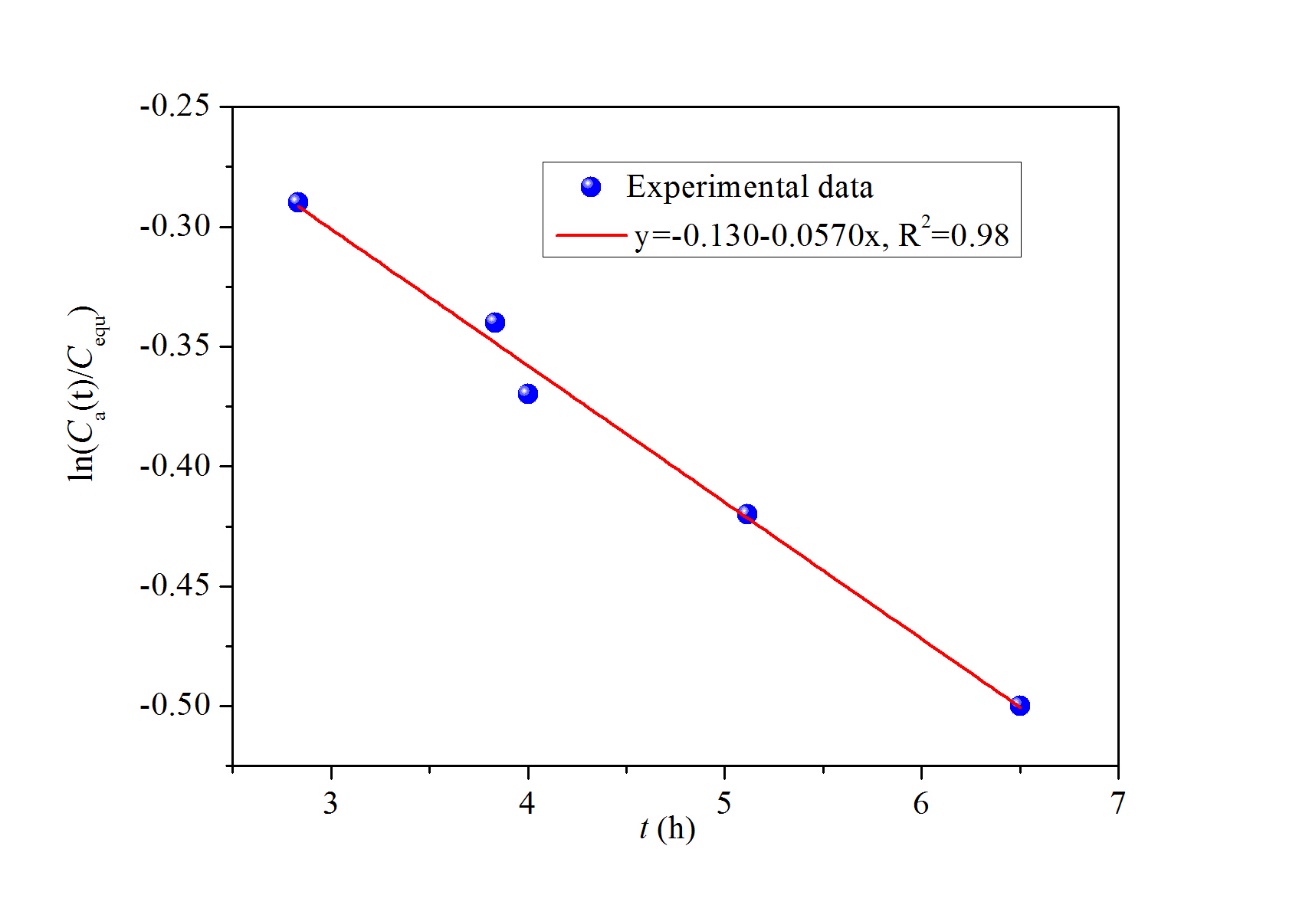


1. Formaldehyde (AH=15.0g/m^3^)


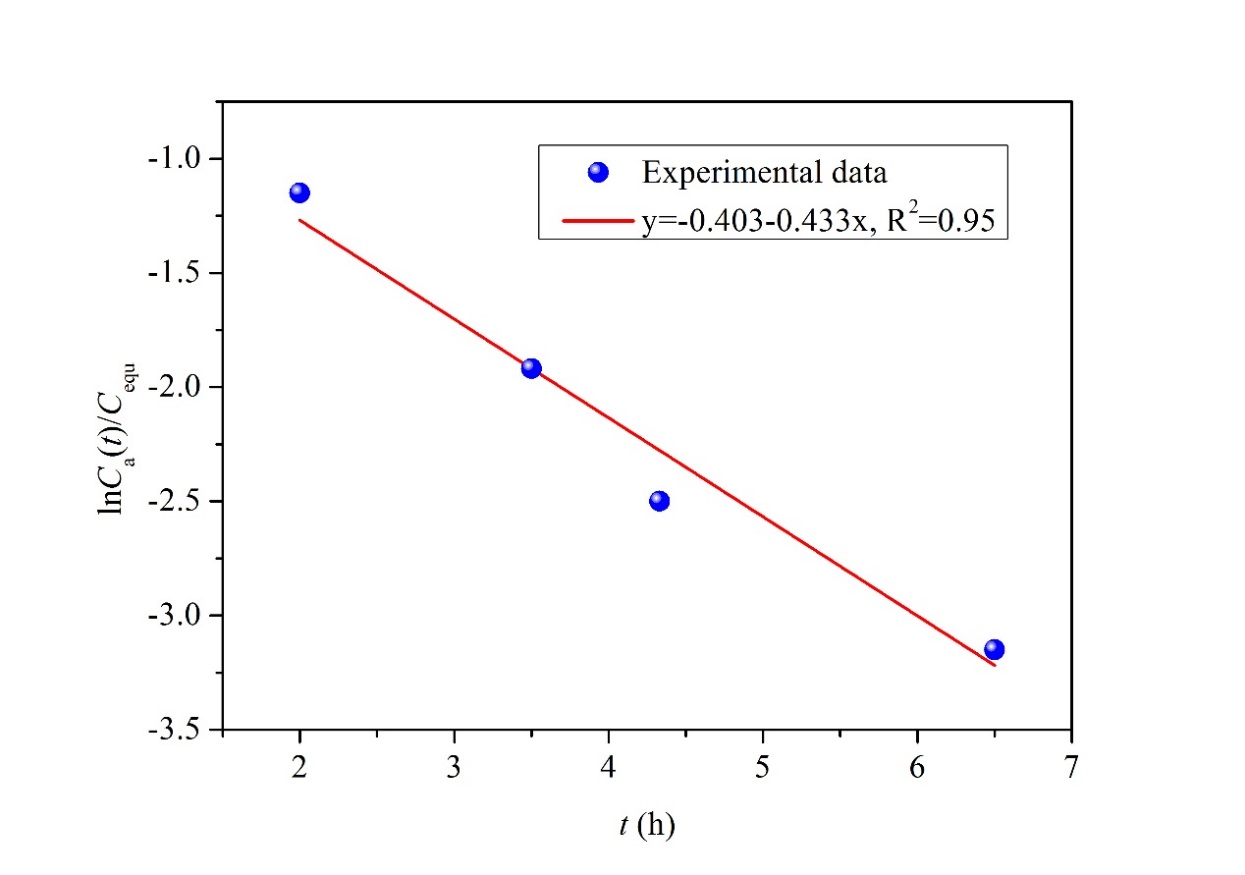


1. Hexaldehyde (AH=9.2g/m^3^)


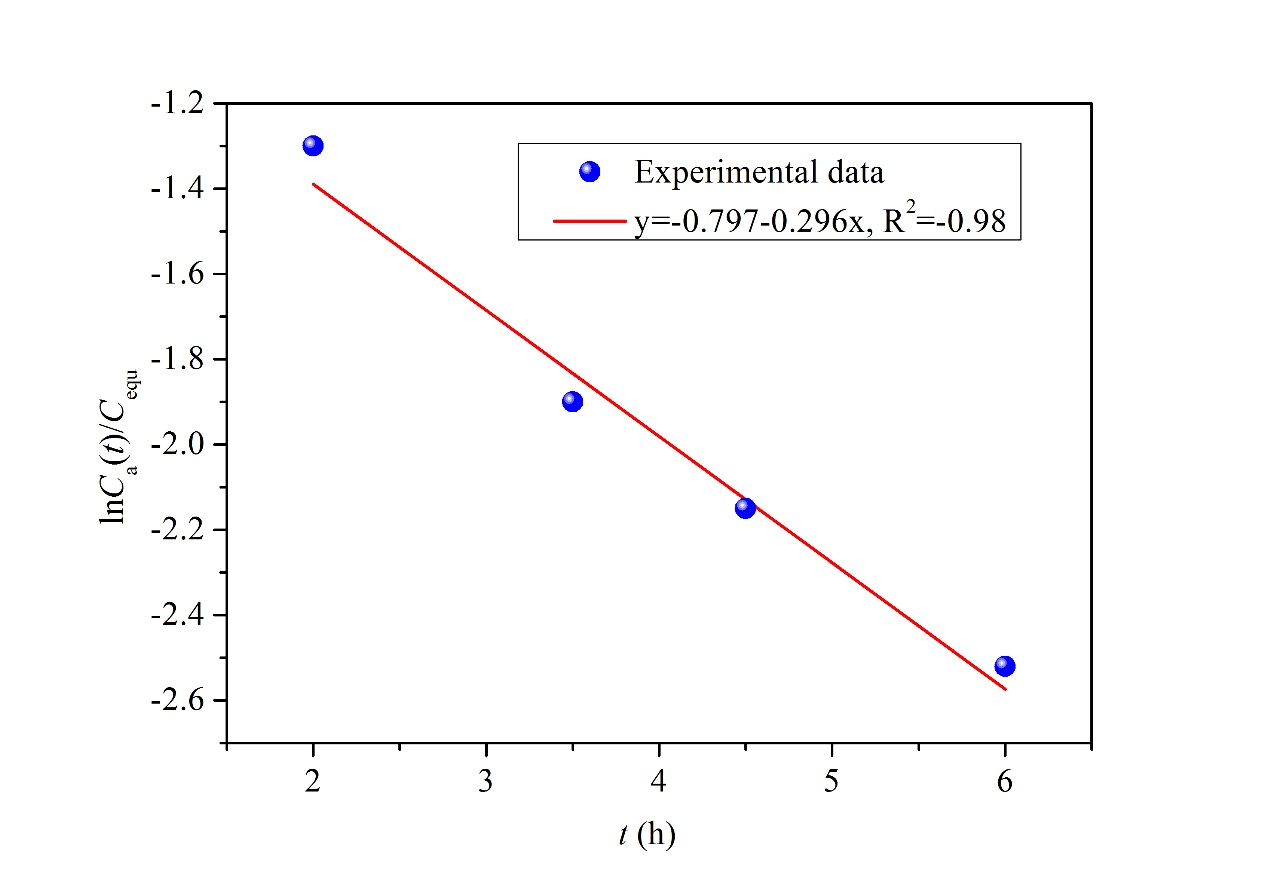


1. Hexaldehyde (AH=12.7g/m^3^)


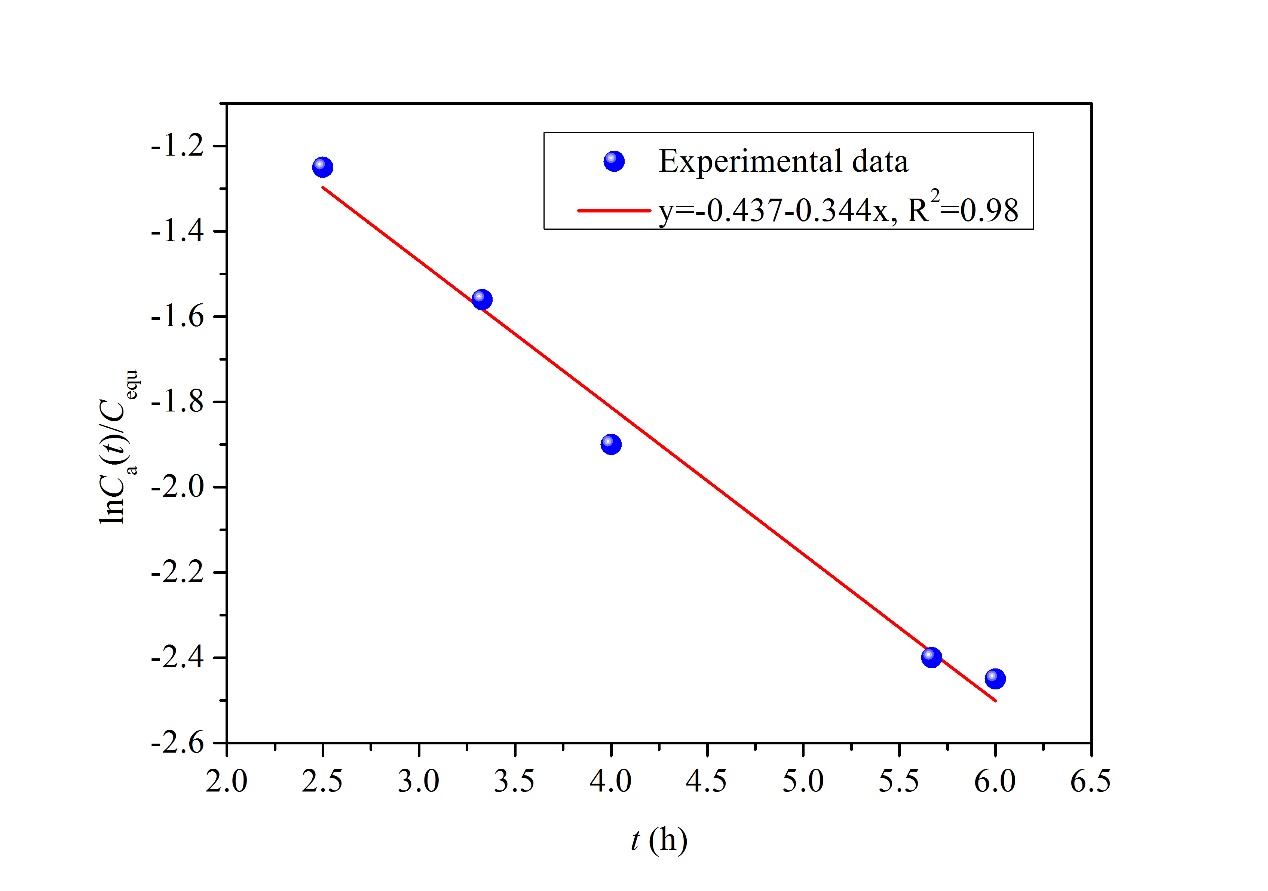


1. Hexaldehyde (AH=15.0g/m^3^)

Fig. S6.


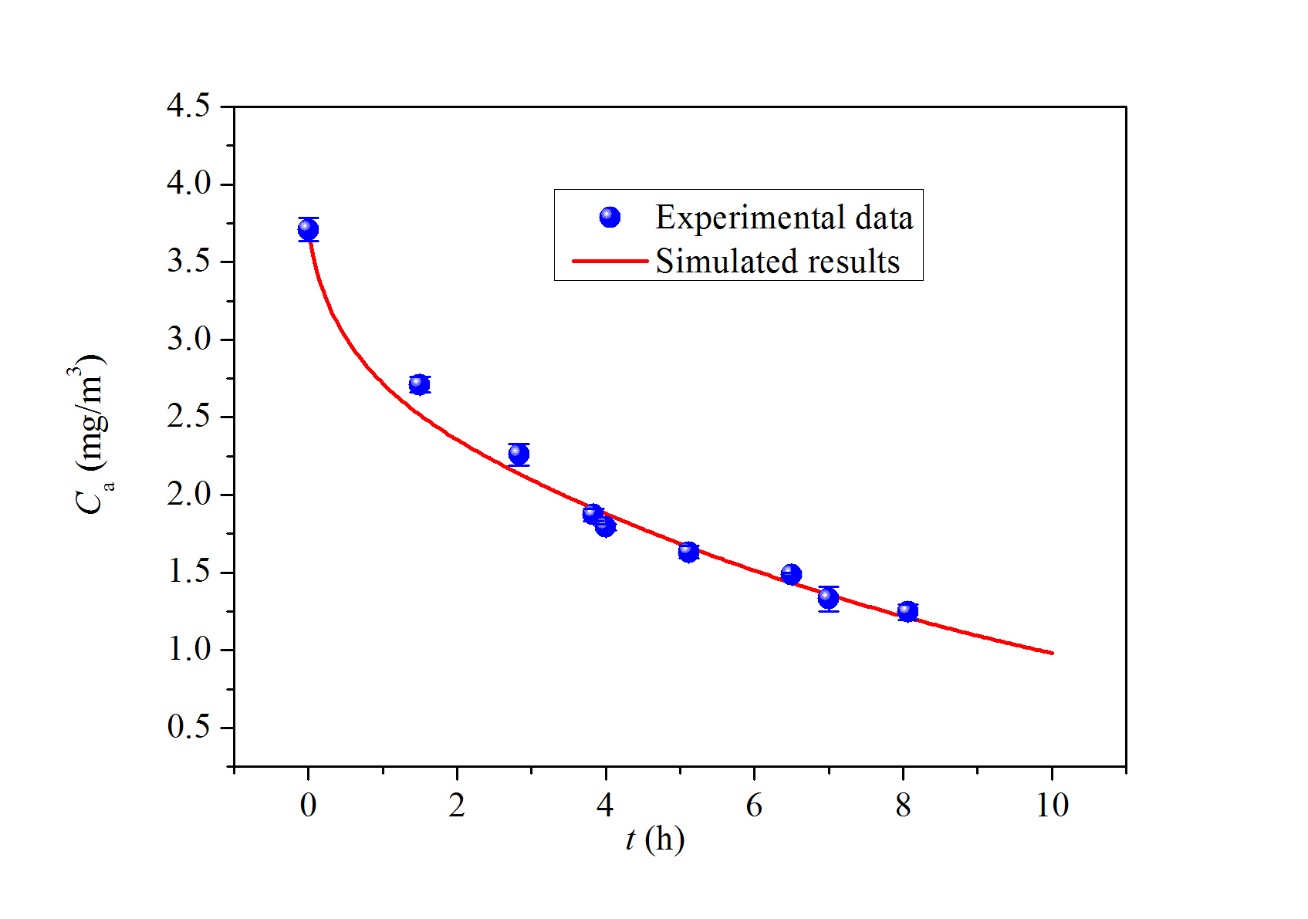


1. Formaldehyde (AH=9.2g/m^3^)


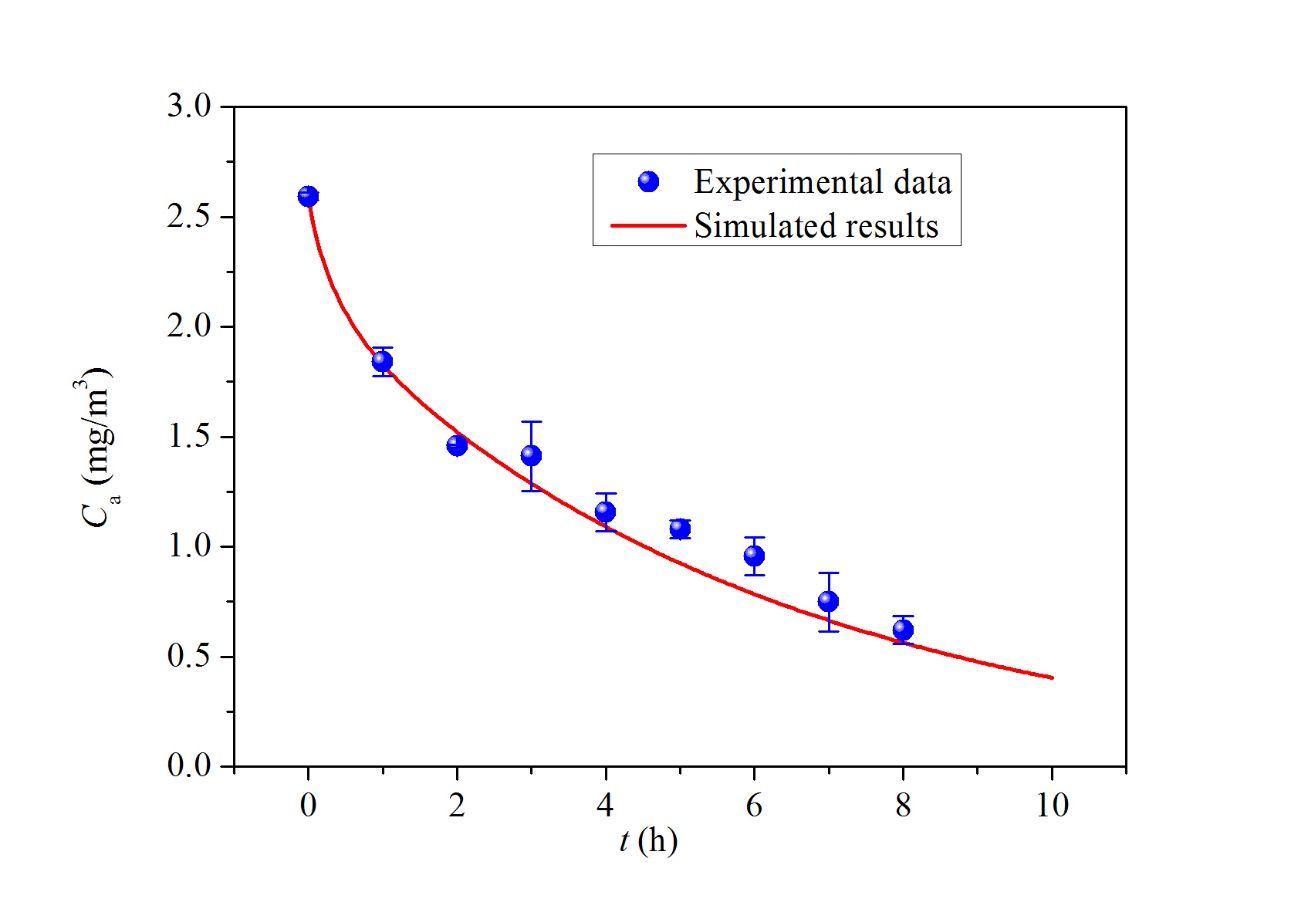


1. Formaldehyde (AH=9.2g/m^3^)


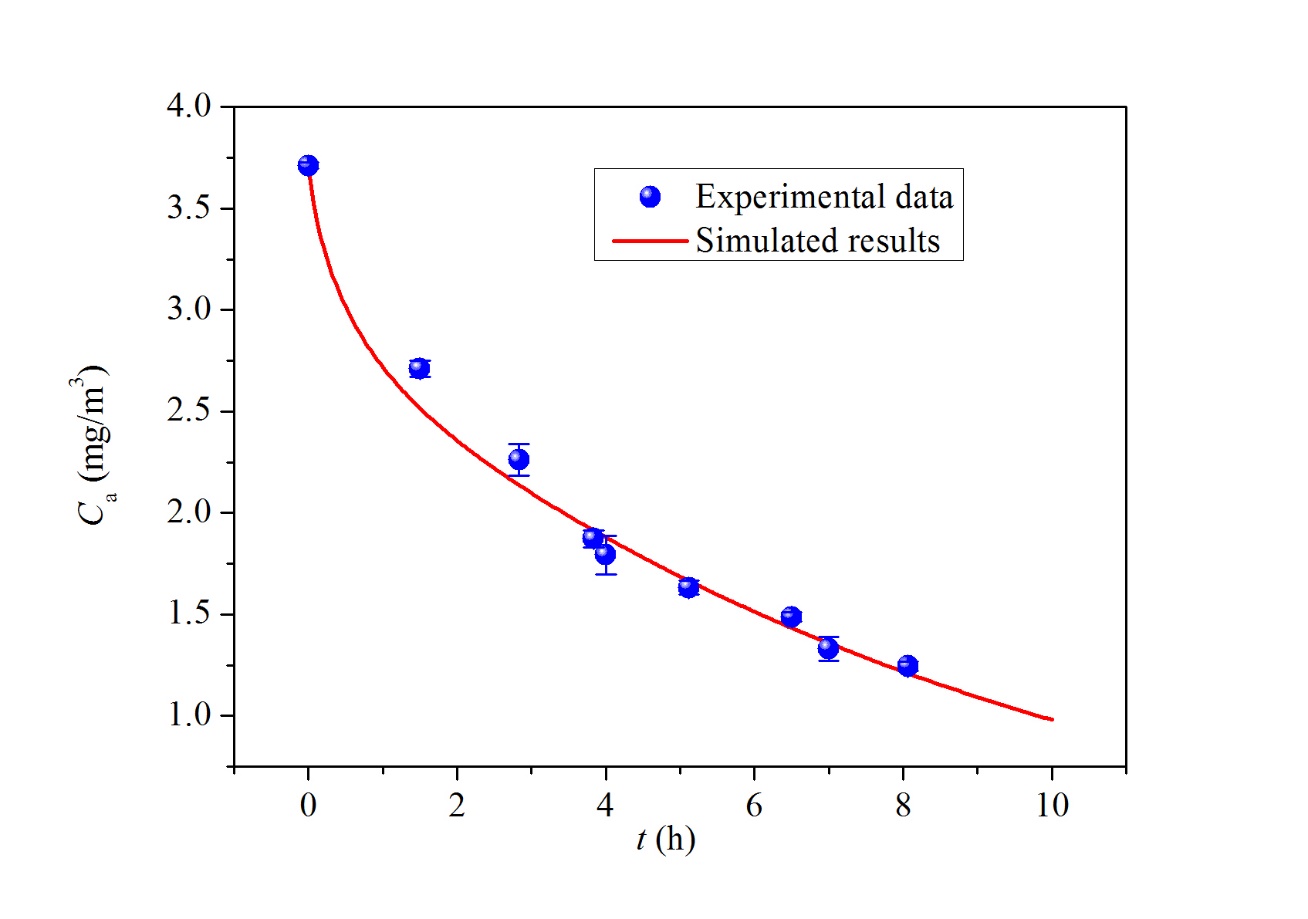


1. Formaldehyde (AH=15.0g/m^3^)


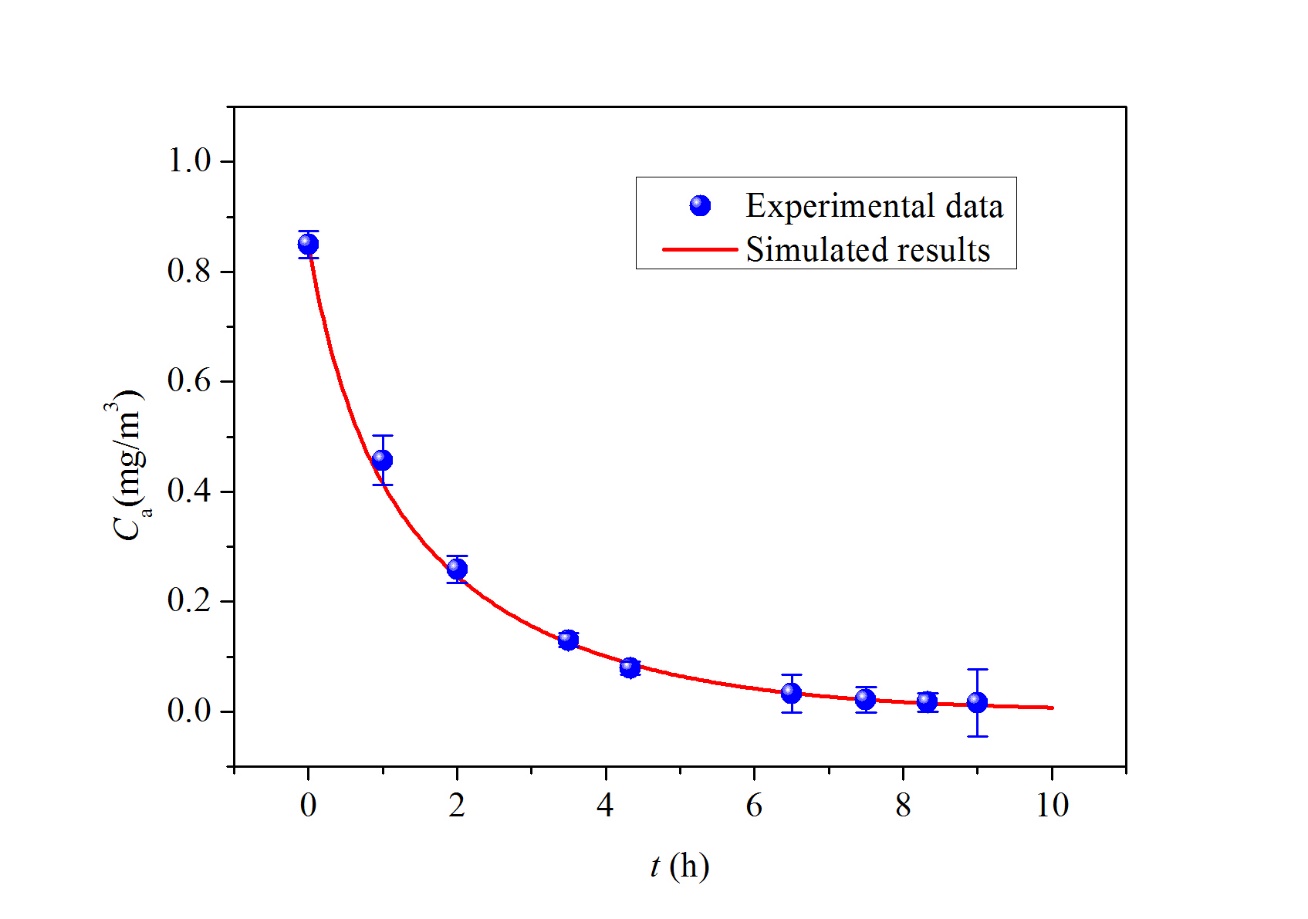


1. Hexaldehyde (AH=9.2g/m^3^)


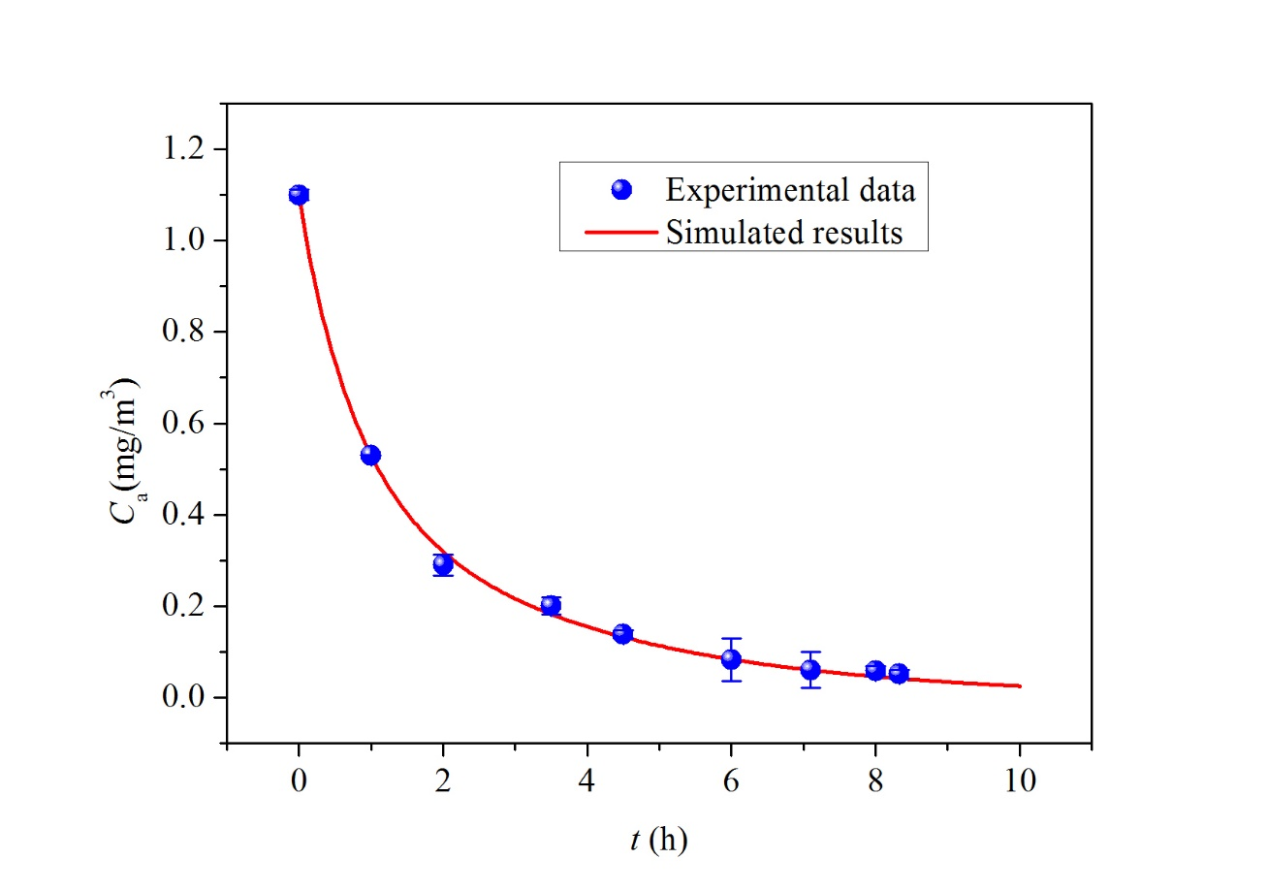


1. Hexaldehyde (AH=12.7g/m^3^)


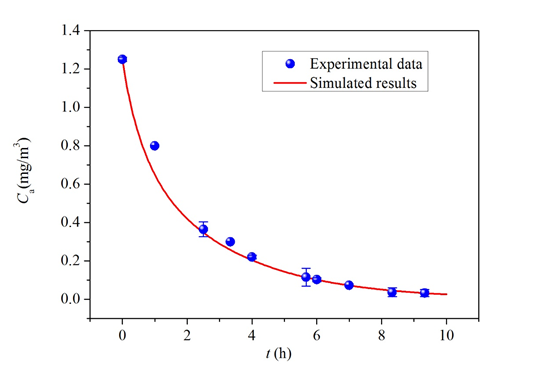


1. Hexaldehyde (AH=15.0g/m^3^)

Fig. S7.


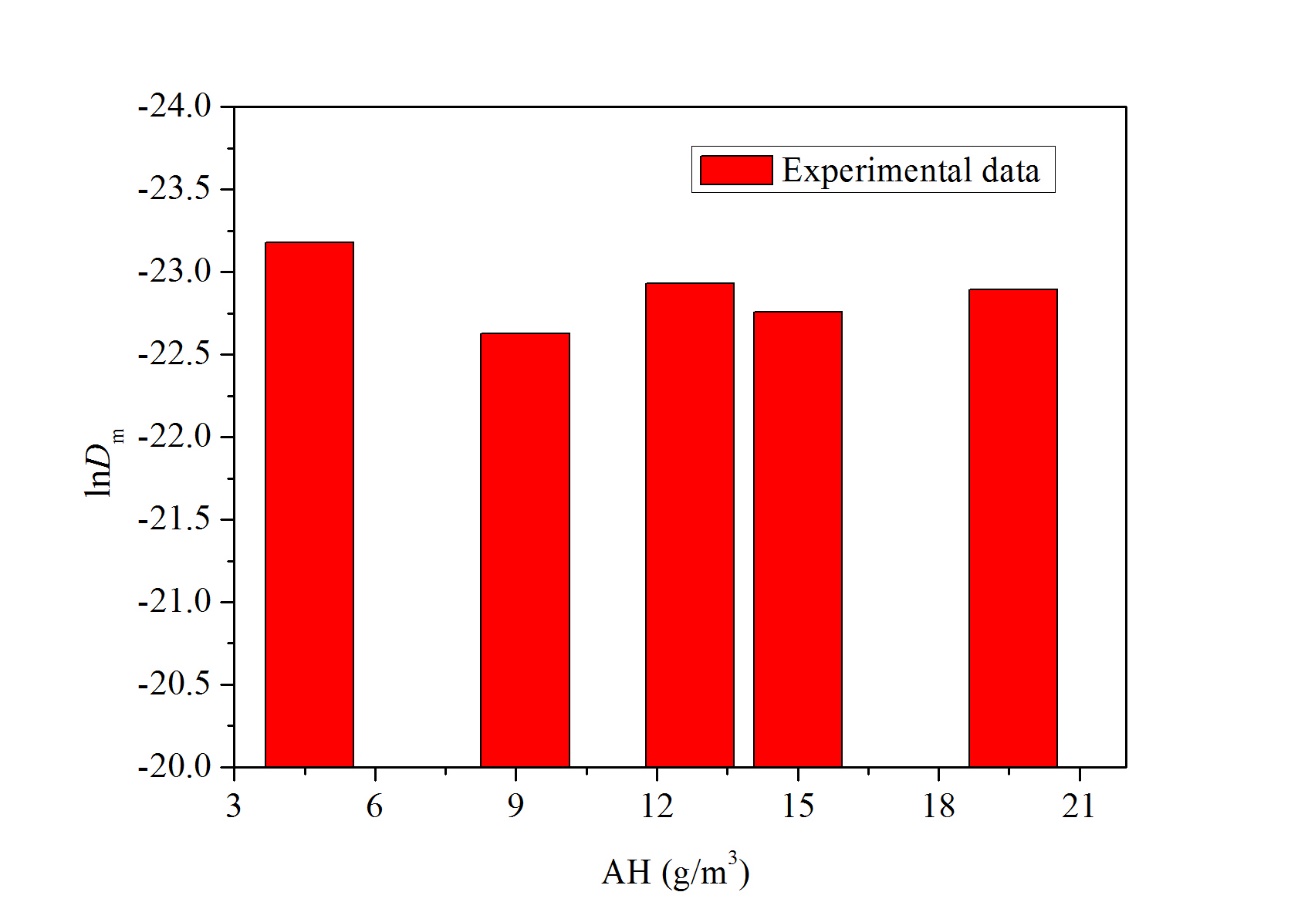


1. Formaldehyde


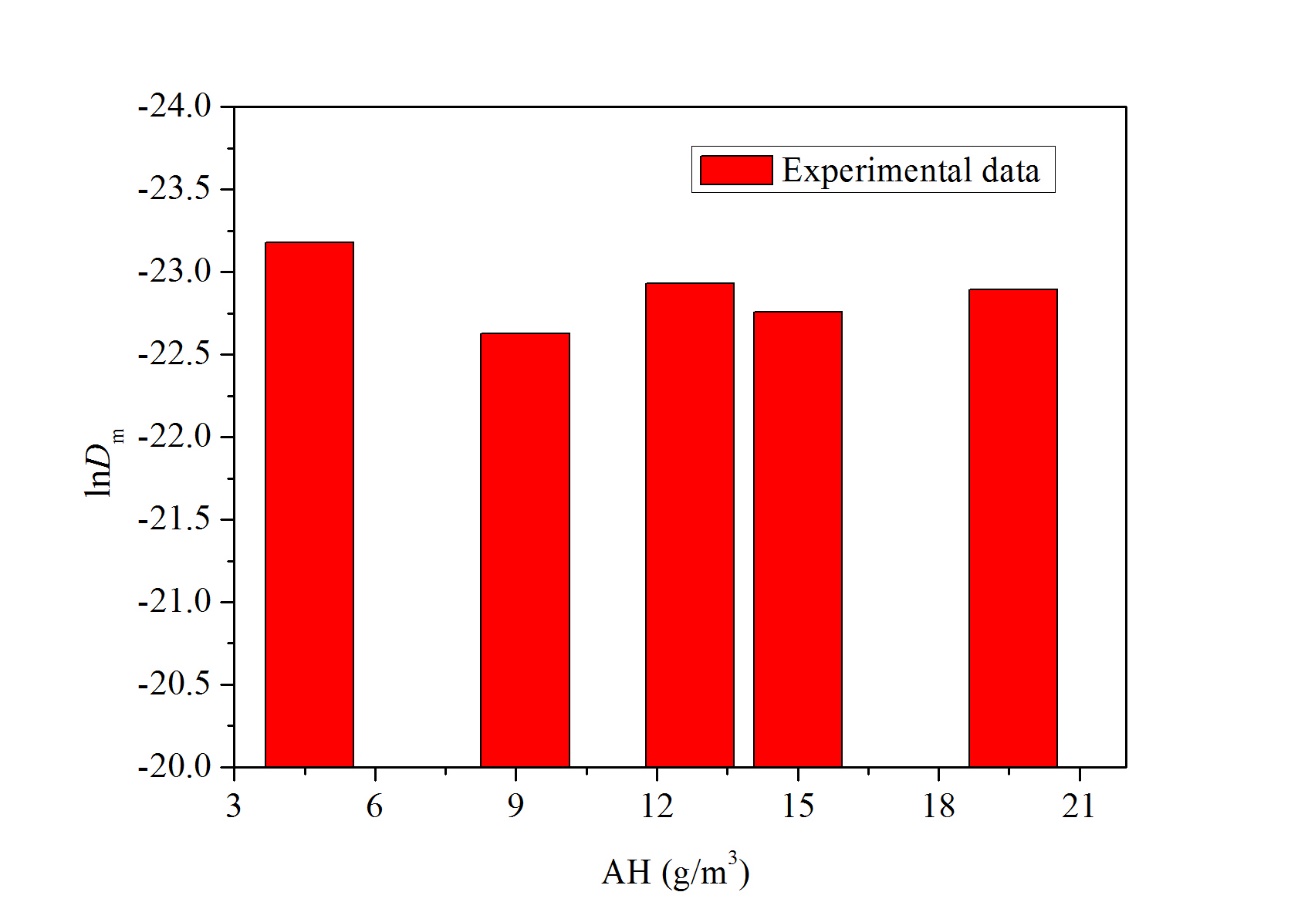


1. Hexaldehyde

Fig. S8.


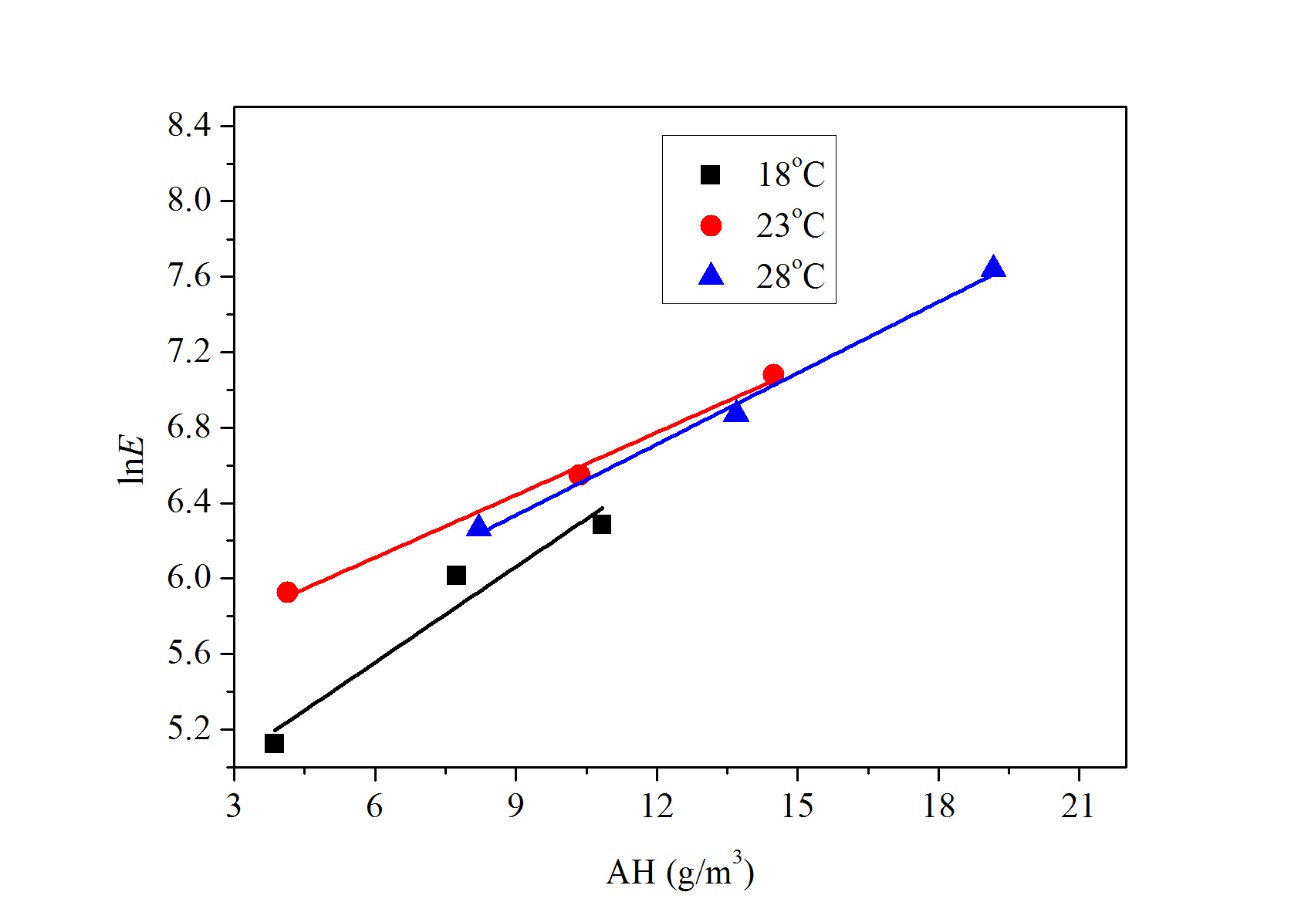


1. 1-methyl-2-pyrrolidion from floor vanish


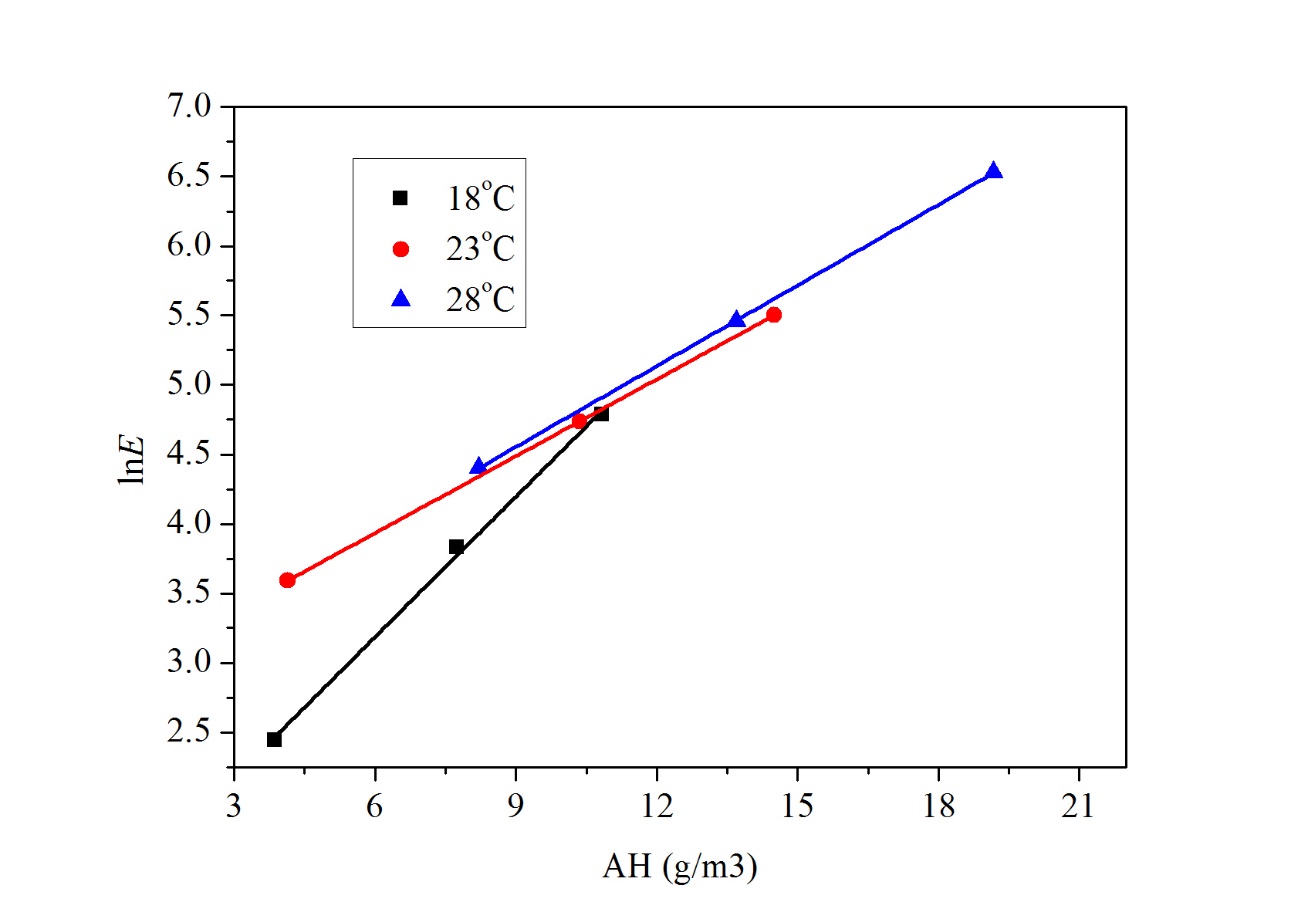


1. butylacetat from floor vanish


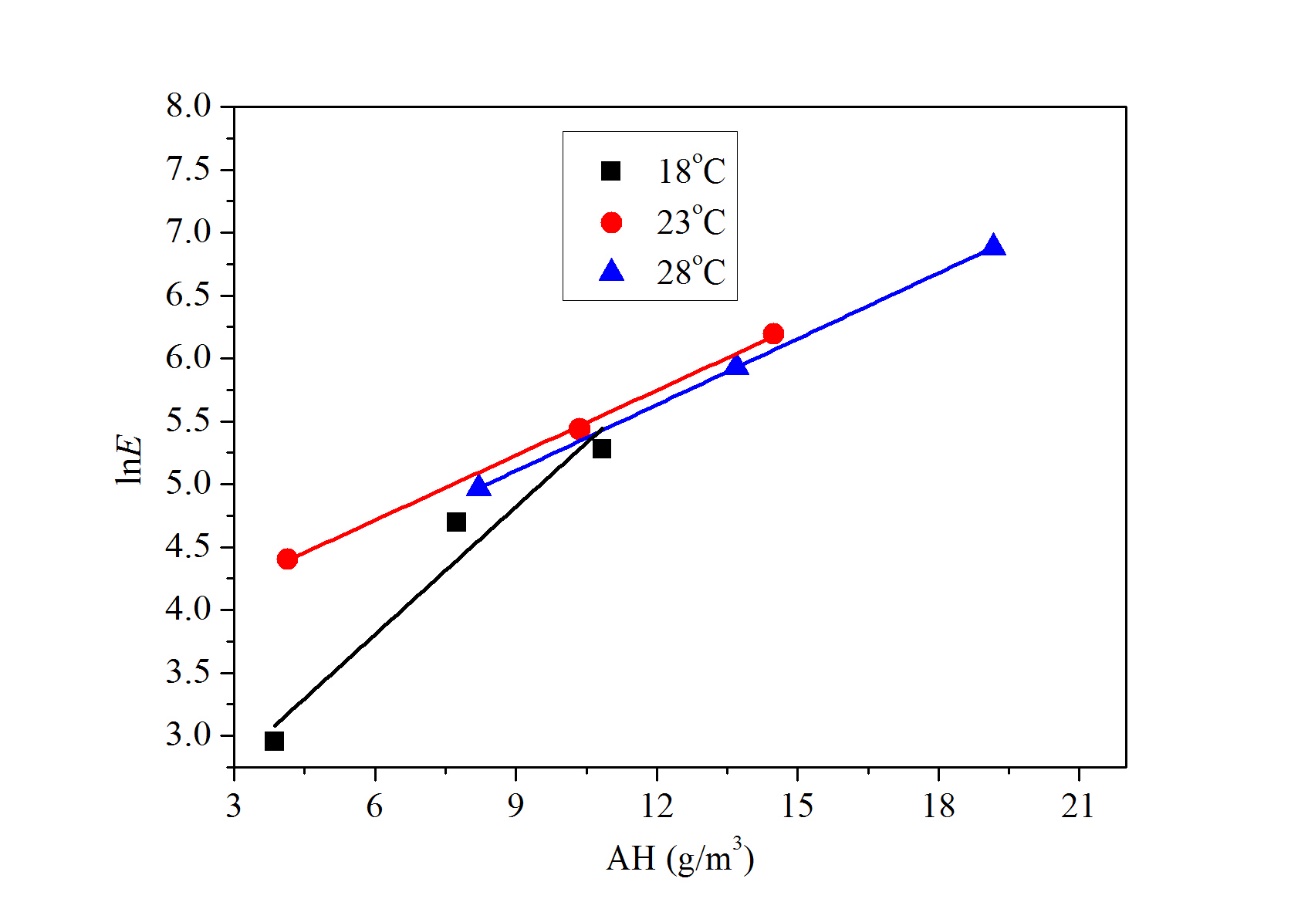


1. 2,(2-ethoxyethoxy)-ethanol from floor vanish


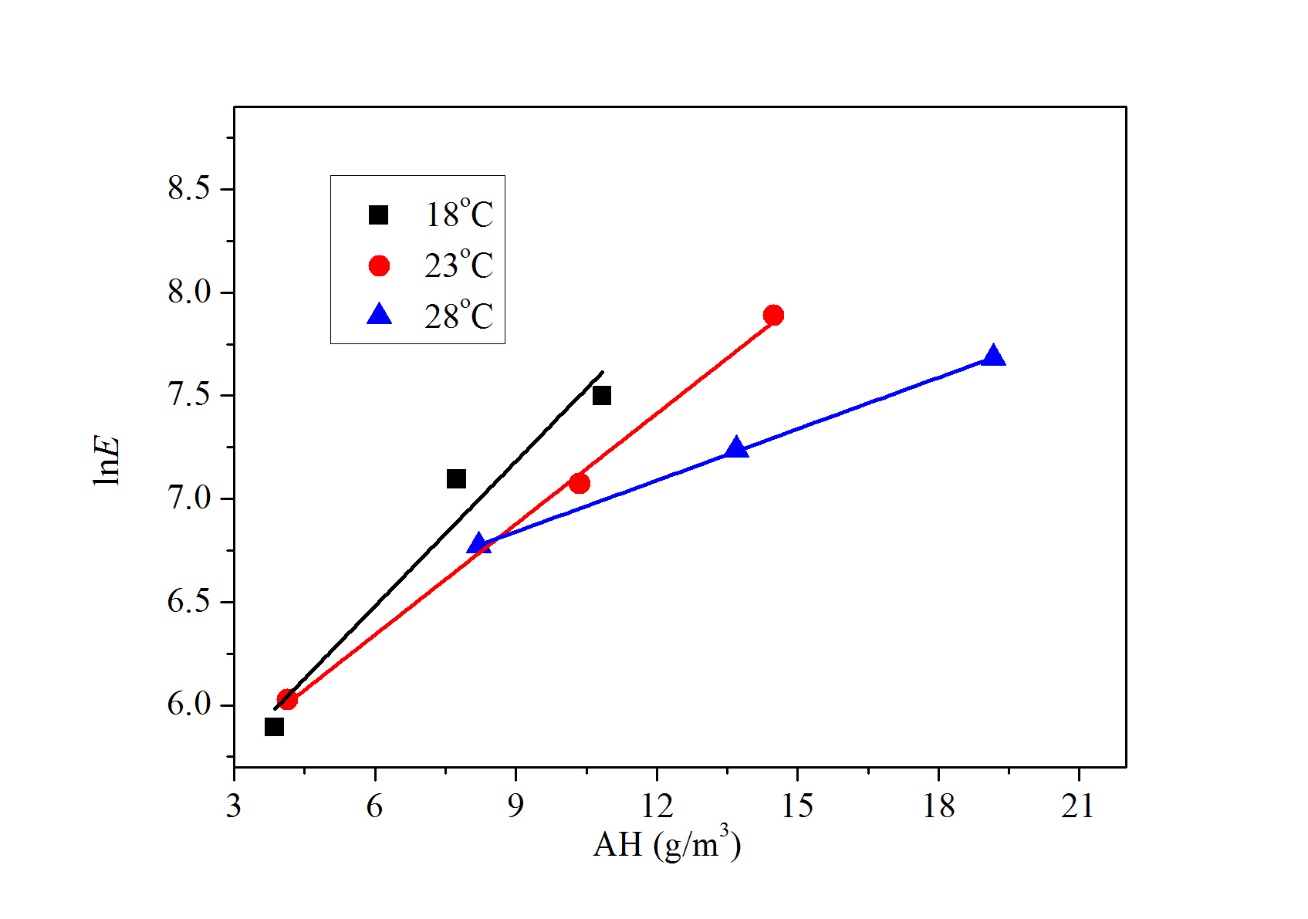


1. 1,2-propandiol from wall paint


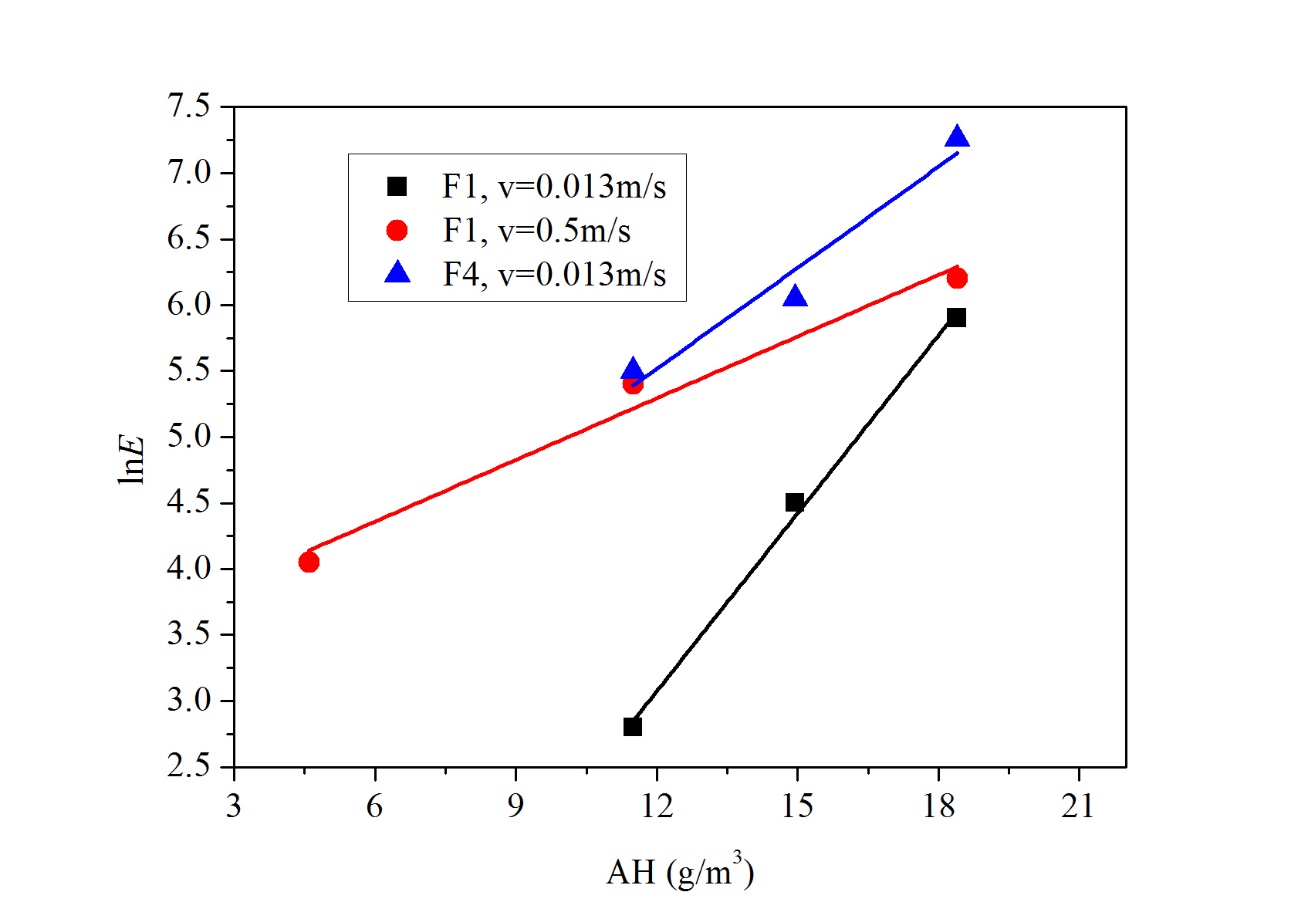


1. formladehyde from filters

**Table S1.** The experimental results of recovery rate in the chamber

| Testing time | *C* (mg/m^3^) | V (L/h) | Tested emission rate (μg/h) | Standard emission rate (μg/h) | Recovery rate (%) |
| --- | --- | --- | --- | --- | --- |
| 1 | 0.826 | 29.1 | 24.024 | 28.350 | 84.9 |
| 2 | 0.778 | 29.4 | 22.879 |  | 80.8 |
| 3 | 0.849 | 28.9 | 24.525 |  | 86.7 |
| 4 | 0.851 | 28.9 | 24.585 |  | 86.9 |
| 5 | 0.790 | 30.4 | 24.008 |  | 84.8 |
| 6 | 0.840 | 30.1 | 25.299 |  | 89.4 |

**Table S2.** Results by treating the experimental data from literature with the derived correlation for emission rate.

| Fang et al.’s experimental data^36^ | | | | | | | | | |
| --- | --- | --- | --- | --- | --- | --- | --- | --- | --- |
| Material | Pollutant | temperature (^o^C) | | *E*_1_ | | *E*_2_ | | R^2^ | |
| Floor vanish | 1-methyl-2-pyrrolidion | 18 | | 0.17 | | 4.54 | | 0.89 | |
|  |  | 23 | | 0.11 | | 5.45 | | 0.99 | |
|  |  | 28 | | 0.13 | | 5.20 | | 0.99 | |
|  | Butylacetat | 18 | | 0.34 | | 1.16 | | 0.99 | |
|  |  | 23 | | 0.18 | | 2.83 | | 0.99 | |
|  |  | 28 | | 0.19 | | 2.81 | | 0.99 | |
|  | 2,(2-ethoxyethoxy)-ethanol | 18 | | 0.34 | | 1.77 | | 0.91 | |
|  |  | 23 | | 0.17 | | 3.68 | | 0.99 | |
|  |  | 28 | | 0.17 | | 3.53 | | 0.99 | |
| Wall paint | 1,2-propandiol | 18 | | 0.23 | | 5.08 | | 0.91 | |
|  |  | 23 | | 0.18 | | 5.27 | | 0.99 | |
|  |  | 28 | | 0.08 | | 6.09 | | 0.99 | |
| Sidheswaran et al.’s experimental data^37^ | | | | | | | | | |
| Material | Pollutant | | Air velocity (m/s) | | *E*_1_ | | *E*_2_ | | R^2^ |
| Filter 1 (F1) | Formaldehyde | | 0.013 | | 0.45 | | -2.32 | | 0.99 |
| Filter 1 (F1) |  |  | 0.5 | | 0.16 | | 3.42 | | 0.96 |
| Filter 4 (F4) |  |  | 0.5 | | 0.26 | | 2.46 | | 0.91 |
